# Polyploidy breaks speciation barriers in Australian burrowing frogs *Neobatrachus*

**DOI:** 10.1371/journal.pgen.1008769

**Published:** 2020-05-11

**Authors:** Polina Yu. Novikova, Ian G. Brennan, William Booker, Michael Mahony, Paul Doughty, Alan R. Lemmon, Emily Moriarty Lemmon, J. Dale Roberts, Levi Yant, Yves Van de Peer, J. Scott Keogh, Stephen C. Donnellan

**Affiliations:** 1 VIB-UGent Center for Plant Systems Biology, Ghent, Belgium; 2 Department of Plant Biotechnology and Bioinformatics, Ghent University, Ghent, Belgium; 3 Division of Ecology & Evolution, Research School of Biology, The Australian National University, Canberra, Australia; 4 Department of Biological Science, Florida State University, Tallahassee, Florida, United States of America; 5 School of Environmental and Life Sciences, University of Newcastle, Callaghan, Australia; 6 Western Australian Museum, Welshpool, Perth, Australia; 7 Department of Scientific Computing, Florida State University, Tallahassee, Florida, United States of America; 8 School of Biological Sciences, and, Centre for Evolutionary Biology, University of Western Australia, Albany, Western Australia, Australia; 9 School of Life Sciences and Future Food Beacon, University of Nottingham, Nottingham, United Kingdom; 10 Bioinformatics Institute Ghent, Ghent University, Ghent, Belgium; 11 Department of Biochemistry, Genetics and Microbiology, University of Pretoria, Pretoria, South Africa; 12 South Australian Museum, North Terrace, Adelaide, Australia; 13 School of Biological Sciences, University of Adelaide, North Terrace, Adelaide, Australia; University of Georgia, UNITED STATES

## Abstract

Polyploidy has played an important role in evolution across the tree of life but it is still unclear how polyploid lineages may persist after their initial formation. While both common and well-studied in plants, polyploidy is rare in animals and generally less understood. The Australian burrowing frog genus *Neobatrachus* is comprised of six diploid and three polyploid species and offers a powerful animal polyploid model system. We generated exome-capture sequence data from 87 individuals representing all nine species of *Neobatrachus* to investigate species-level relationships, the origin and inheritance mode of polyploid species, and the population genomic effects of polyploidy on genus-wide demography. We describe rapid speciation of diploid *Neobatrachus* species and show that the three independently originated polyploid species have tetrasomic or mixed inheritance. We document higher genetic diversity in tetraploids, resulting from widespread gene flow between the tetraploids, asymmetric inter-ploidy gene flow directed from sympatric diploids to tetraploids, and isolation of diploid species from each other. We also constructed models of ecologically suitable areas for each species to investigate the impact of climate on differing ploidy levels. These models suggest substantial change in suitable areas compared to past climate, which correspond to population genomic estimates of demographic histories. We propose that *Neobatrachus* diploids may be suffering the early genomic impacts of climate-induced habitat loss, while tetraploids appear to be avoiding this fate, possibly due to widespread gene flow. Finally, we demonstrate that *Neobatrachus* is an attractive model to study the effects of ploidy on the evolution of adaptation in animals.

## Author summary

Polyploidy or whole genome duplication is rare in animals and usually polyploid animals reproduce asexually. The Australian burrowing frogs of the genus *Neobatrachus* form an interesting exception amongst vertebrates with multiple independently originated autotetraploid sexual species. We generated population genomic data from 87 animals representing all six diploid and three tetraploid species of *Neobatrachus*. We show that, while diploid *Neobatrachus* species seem to be isolated from each other, their sister tetraploid species experience substantial levels of gene flow, and have wider distributions. Furthermore, we observe asymmetric gene flow from diploids to tetraploids. Based on our genomic and climate analyses we suggest that such inter-specific hybridization mediated by whole genome duplication rescues species diversity and allows tetraploids to more easily avoid impacts of climate-induced habitat loss.

## Introduction

Polyploidy or whole genome duplications (WGDs) play important roles in ecology and evolution [1, 2]. Although polyploidization predominantly occurs in plants, polyploidy has also played an important role in animal evolution. For instance, two ancient WGDs occurred early in the vertebrate lineage [3], while more recent WGDs occurred in several animal groups, including insects, molluscs, crustaceans, fishes, amphibians and reptiles [4–6]. The majority of polyploid animals switch to diverse modes of asexual reproduction after polyploid formation [4, 7, 8]. Amphibians, and more specifically anurans, are among very few exceptions exhibiting multiple independent occurrences of diploid and sexually reproducing polyploid sister species [9]. The most famous example is probably the clade of model frogs *Xenopus* which is enriched with allopolyploids [10, 11]. Overall, polyploid occurrences in amphibia have been described in at least 15 different families [12], which makes it the most frequent among sexually reproducing vertebrates, possibly due to homomorphic (undifferentiated) sex chromosomes [13] which do not require dosage compensation [14].

Here, we focus on a group of widely distributed, endemic, Australian burrowing frogs: *Neobatrachus*. This genus comprises six diploid (*N*. *albipes*, *N*. *fulvus*, *N*. *pelobatoides*, *N*. *pictus*, *N*. *sutor*, *N*. *wilsmorei*; 2n = 24) and three tetraploid (*N*. *aquilonius*, *N*. *kunapalari*, *N*. *sudellae*; 4n = 48) species [15, 16], all characterised by bisexual reproduction [17]. Taxonomic status of a previously described tetraploid species *N*. *centralis* has been redefined and synonymized with *N*. *sudelli* [15], which was later changed to a version with fenimine termination *N*. *sudellae*, as the species was named after a woman, Miss J. Sudell of Warwick [18]. Tetraploid species of *Neobatrachus* were suggested to have independent origins based on mitochondrial DNA (mtDNA) [19]. At least one of the tetraploid species—*N*. *sudellae*—was suggested to have originated through autotetraploidy rather than allotetraploidy, as they exhibit tetrasomic inheritance and show a prevalence of tetravalent over bivalent formations during meiosis [17]. *Neobatrachus* species are well defined based on external morphology, male advertisement calls and divergence at allozyme loci [20–22]. Generally, frog call structure differs among ploidies with higher ploidy species having lower pulse rates, a trait linked to nuclear volume increase with increasing ploidy [23]. Indeed, tetraploid *Neobatrachus* species have lower pulse number and rate in their advertisement calls compared to diploids with multiple pulses in their calls (however *N*. *sutor* (2n) and *N*. *wilsmorei* (2n) have calls with a single pulse). However, each of the *Neobatrachus* species retain distinct calls [24, 25]. This differs from the more extensively studied gray treefrog, *Hyla versicolor* [26, 27], where tetraploids may have also originated from multiple independent origins but have calls that are largely similar across lineages [28]. The primary aim of our study is to resolve the phylogenetic relationships and reticulation of the *Neobatrachus* species and describe the evolutionary origin of the tetraploid *Neobatrachus* lineages.

While polyploidization has occurred frequently across the tree of life, the evolutionary benefits of WGDs remain elusive. Polyploidy has been associated with greater tolerance to harsher conditions, but it is not clear whether WGDs broadly provide a fitness advantage or are simply a consequence of elevated rates of unreduced gamete formation [2, 29–31], which might be more prone to occur in extreme environments. Polyploids of hybrid origin (allopolyploids) may benefit from heterosis due to increased genetic variation, instantaneous shifts into intermediate or new ecological niches, and the redundancy of independently segregating gene copies [2, 29]. However, newly formed polyploids are simultaneously subject to several disadvantages perhaps most prominent of which is their low abundance compared to the established non-polyploids [29]. Polyploids face strong frequency dependent selection because they are unlikely to produce viable or fertile offspring if crossed with a diploid. Therefore, rare polyploid types are at a disadvantage. Conversely, while autopolyploids retain many of the same disadvantages as allopolyploids, the advantages of autopolyploidy are much less clear [32]. Recent example of possible advantage of autopolyploidy in frogs comes from *Odontophrynus* species [12, 33–35], where polyploids exhibited higher stress response and lower nuclear abnormalities compared to the sister diploids coexisting in the same agroecosystems [36]. In *Neobatrachus*, while tetraploid species are distributed sympatrically with some of the diploid species, they are also able to occupy more arid areas across Australia [2, 19]. Here, using a combination of climate, occurrence and genomic data, we test whether *Neobatrachus* polyploids occupy larger or different niches and whether they may have a greater genetic adaptive potential in changing environments. The latter becomes critically important, since changing environments drive amphibian extinction rates, which continue to increase and are comprised of many interdependent factors such as habitat loss, emergence and spread of diseases, invasive species and pollution [37–40].

In the current study, we use an anchored hybrid enrichment approach (AHE) [41–44] to resolve the phylogenetic relationships among *Neobatrachus* species, and to assess fine-scale intra-specific genetic population structure. We also quantify the extent of hybridization between the nine *Neobatrachus* species with a particular focus on taxa with contrasting ploidies. Finally, we combine population dynamics assessments with changes in ecologically suitable areas for each species to describe population responses to climate changes.

## Results

### Evolutionary history of *Neobatrachus*

**Fig 1:**
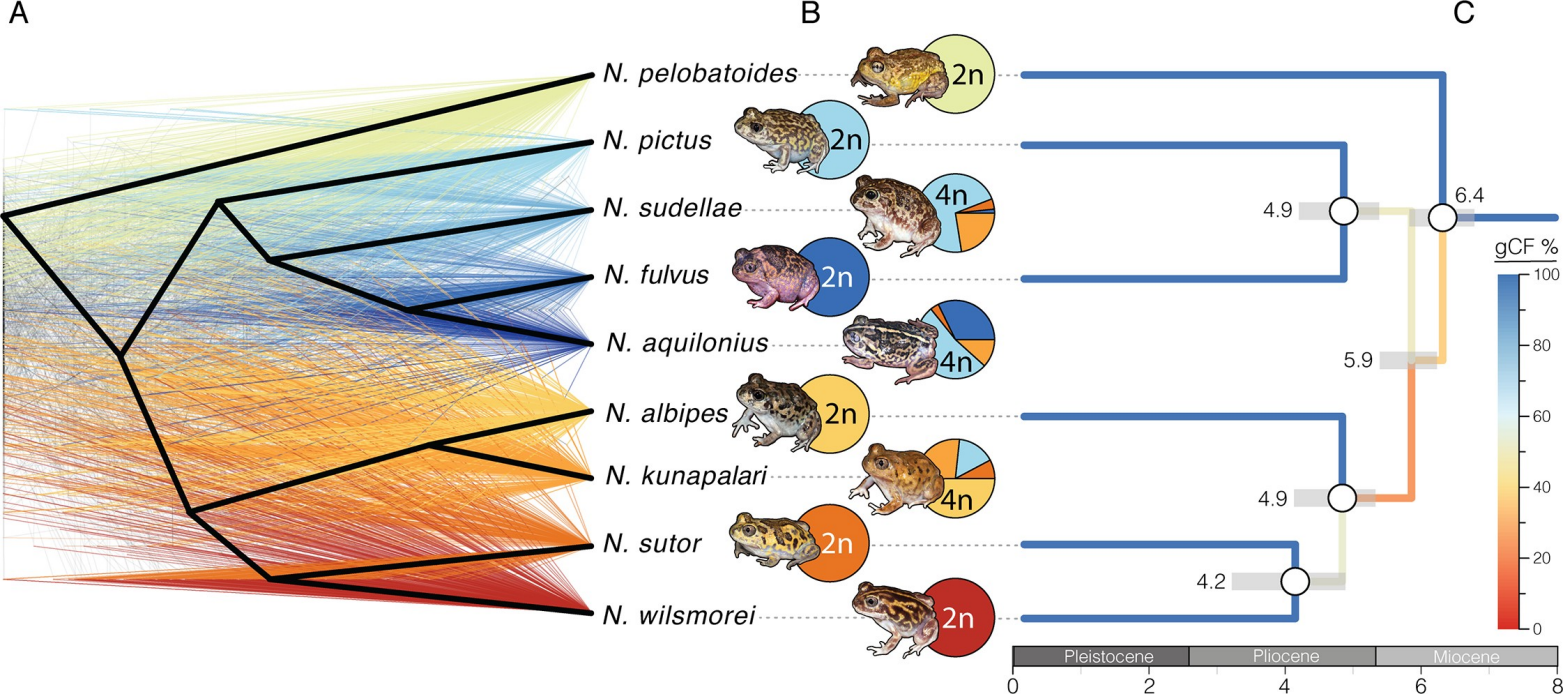
Independent origins of *Neobatrachus* tetraploids and high levels of reticulation. (A) Gene trees, colored by clade, for 361 nuclear loci based on 2 individuals per species show considerable incongruence and differ from the species trees (bold black topology). Conflict between gene tree clusters (S3 Fig) and the nuclear species tree suggest non-bifurcating relationships between the species. (B) Pie charts represent summarised admixture proportions for each species (summing assignments for each individual, S1 Fig, Fig 2) at optimal clustering with K = 7. Tetraploids (*N*. *sudellae*, *N*. *aquilonius* and *N*. *kunapalari*) show highly admixed ancestries. (C) Dated diploid-only species tree. Colors represent consistency levels between gene genealogies with red being most conflicted and blue most consistent. Grey bars represent 95% confidence intervals on the ages of nodes, noted in millions of years before present.

**Fig 2:**
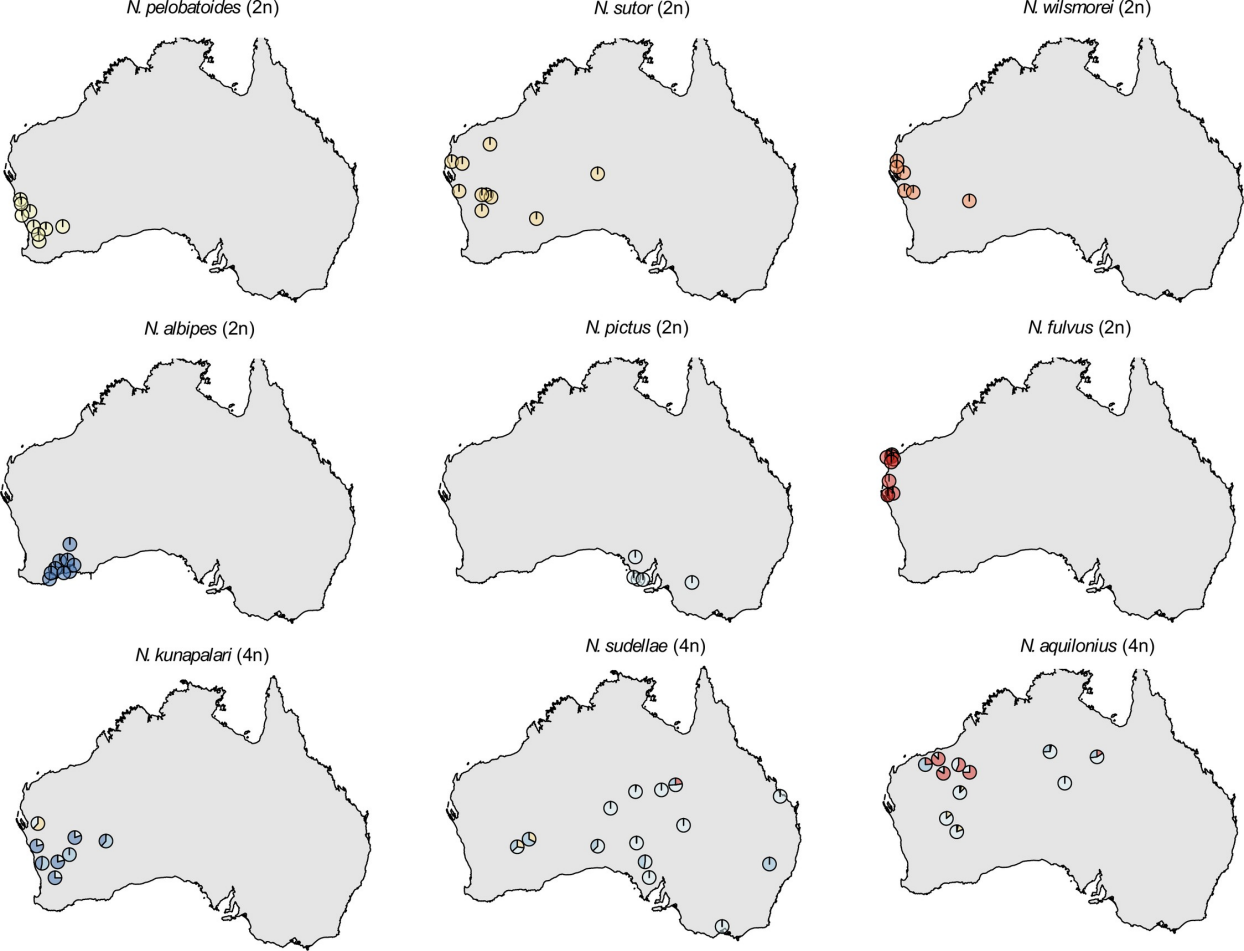
ADMIXTURE results (K = 7) shown separately for each species. According to the geographical locations of the sampled individuals, pie charts show the probability of the assignment of the individual to one of the 7 individually colored clusters. Overlapping pie charts on the map have been moved just enough to appear separate. Diploid *Neobatrachus* species (top 6: *N*. *pelobatoides*, *N*. *albipes*, *N*. *wilsmorei*, *N*. *sutor*, *N*. *pictus*, *N*. *fulvus*) are each assigned to separate clusters, while all three tetraploid species (bottom 3: *N*. *kunapalari*, *N*. *sudellae*, *N*. *aquilonius*) show inter-species admixture.

We generated sequence data and alignments for 439 targeted orthologous nuclear loci of 87 *Neobatrachus* individuals spanning the entire genus as well as nine *Heleioporus* individuals as outgroups (see Methods). We filtered out six individuals which did not meet our missing data threshold and six individuals where estimated ploidy did not correspond to the expected (see Methods and S1 Table). Our further analysis includes 75 *Neobatrachus* individuals: 8 *N*. *albipes* (2n), 8 *N*. *fulvus* (2n), 7 *N*. *pelobatoides* (2n), 5 *N*. *pictus* (2n), 9 *N*. *sutor* (2n), 5 *N*. *wilsmorei* (2n), 14 *N*. *sudellae* (4n), 11 *N*. *aquilonuis* (4n), 8 *N*. *kunapalari* (4n). We then built a species tree and gene trees from the sequenced loci with ASTRAL-II [45] using RaxML [46] (Fig 1A). This revealed extensive conflict between gene genealogies and the species tree (Fig 1A, S1 Fig and S2 Fig). Multidimensional scaling (MDS) of gene tree topologies suggested that nuclear loci constitute either two or four topology clusters (S3 Fig), indicating competing signal. To investigate the population structure of *Neobatrachus* further we assessed it by ADMIXTURE [47] on extracted polymorphism data (66,789 sites in total; see *Methods*; Fig 1B, Fig 2, S1 Fig, S4 Fig). Overall, admixture clustering corresponded with the phylogenetic placement of the individuals on the species tree (Fig 1 and S1 Fig).

We first focus on the evolutionary history of the diploid *Neobatrachus* species. Diploids were clearly split at K = 7 and did not show admixed individuals (Fig 1, Fig 2 and S1 Fig). However, the gene genealogies from diploid species only also show inconsistency among each other (Fig 1C and S5 Fig). To distinguish between the scenarios of (1) rapid speciation and (2) possible incomplete lineage sorting (ILS) and/or gene flow between the diploid species we estimated genealogies conflict within loci by randomly sampling individuals representing each species. If there has been rapid speciation we expect to see consistent genealogies within each locus regardless of individual chosen to represent each species, but in case of ILS and/or gene flow gene genealogies within one locus should remain in conflict with each other. We found that within locus genealogies are consistent with each other and conflict remains only between loci genealogies (S6 Fig), which supports the scenario of rapid speciation of the diploid species without secondary contacts or persistent incomplete lineage sorting. This is also consistent with clear ADMIXTURE clustering of the diploids (Fig 1B). In the absence of informative fossil material, we estimated the approximate evolutionary timescale of the *Neobatrachus* diploid species divergence (Fig 1C) using secondary calibrations [48]. Interspecific divergence times provided support for a relatively old (older than 4 Mya) origin of *Neobatrachus* species (Fig 1C), which also argues in favor of rapid speciation rather than ILS explaining gene tree inconsistencies.

The evolutionary history of the tetraploid *Neobatrachus* species is more obscure. However, despite the conflict between gene genealogies and the species tree, they all demonstrate that the three tetraploid species do not form a monophyletic group (Fig 1A). All three tetraploid species showed admixture with each other and with local diploid species (Fig 1B, Fig 2 and S1 Fig). The differential assignment of each tetraploid individual within the species (the discrepancies between the pie-charts on Fig 2) argues in favour of an autopolyploid (non-hybrid) origin with sequential unequal gene flow rather than an allopolyploid (hybrid) origin (in this case admixture assignments would show consistent mixed assignment of the tetraploid individuals to both parental species).

In order to distinguish more clearly between allo- and autopolyploid origin of the tetraploids we modeled expected allele frequency distributions for allo- and autotetraploids to estimate the inheritance mode (see Methods). As previously shown [49, 50] allotetraploid individuals with disomic inheritance mode are expected to have an excess of intermediate frequency alleles (AABB) rather than rare alleles (ABBB or AAAB). We simulate expectation of the biallelic allele frequencies for autotetraploids by combining in a pairwise manner the alignments of the diploid individuals within the species, when combination of the diploids between the species simulates expectations for allotetraploids. The allele frequencies distributions for *N*.*sudellae* (4n) and *N*. *aquilonius* (4n) did not have the excess of intermediate alleles expected for allotetraploids and seem to correspond to an autotetraploid origin (S8 Fig, see Methods). *N*. *kunapalari* showed a mixed inheritance pattern, so we could not reject either allo- or autopolyploid origins for this species. However, the most parsimonious explanation of such a pattern is an autotetraploid origin with later extensive gene flow from a different (non-parental diploid) or a different autotetraploid species. Overall, our results seem to support the previously suggested autotetraploid origin of *Neobatrachus* tetraploid species [17, 19].

**Fig 3:**
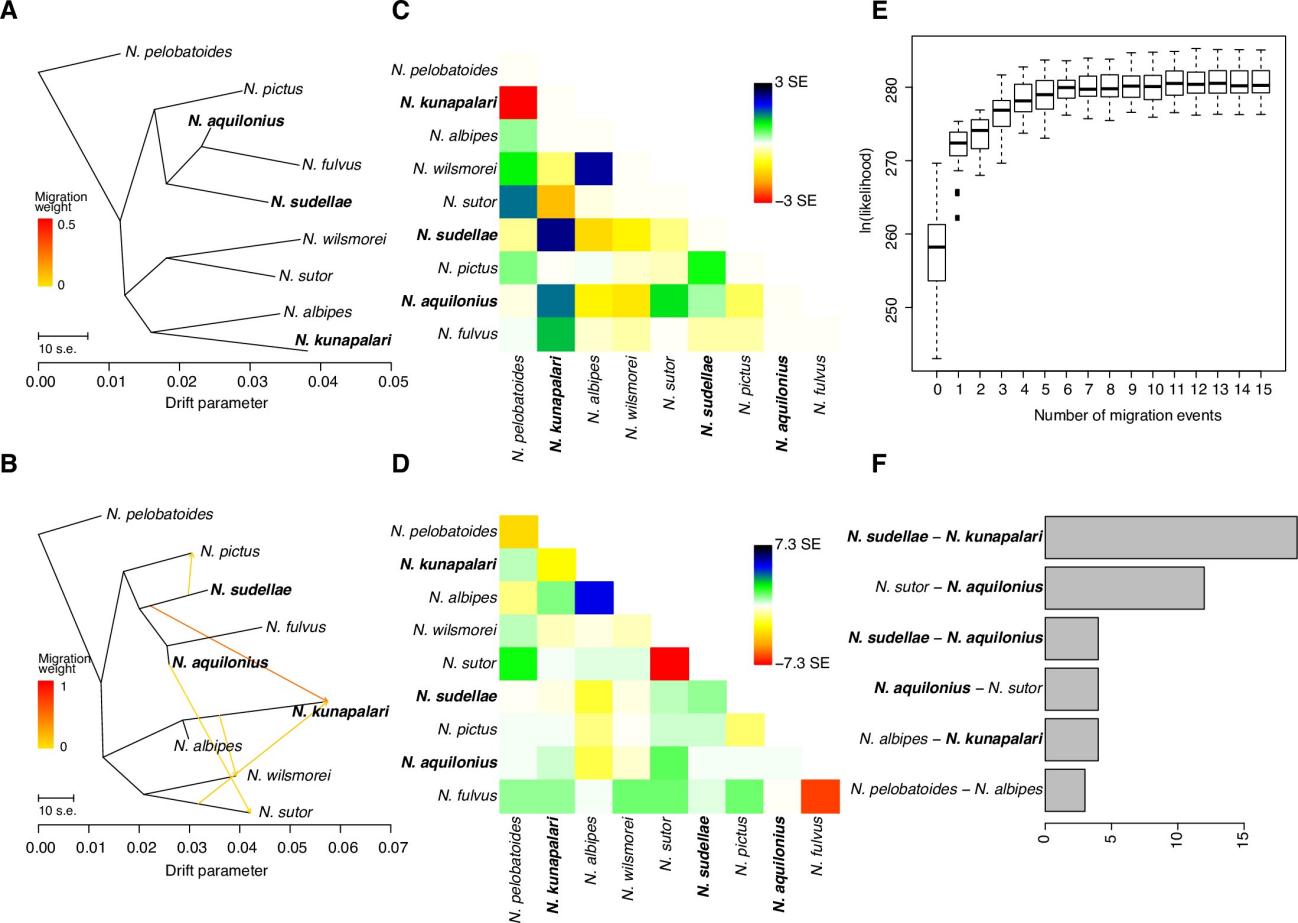
Widespread introgression between *Neobatrachus* species. (A) Bifurcating maximum likelihood tree produced by TreeMix. (B) Example of a graph produced by TreeMix with 5 allowed migration events. (C) Scaled residual fit between observed data and predicted model in (A). Plot shows half of the residual covariance between each pair of populations divided by the average standard error across all pairs. Positive residuals represent populations where the model underestimates the observed covariance, meaning that populations are more closely related to each other in the data than in the modeled tree. Such population pairs are candidates for admixture events. Similarly, negative residuals indicate pairs of populations where the model overestimates the observed covariance. Overall, the residual plot of the model suggested that model fit could be improved by additional edges (migration events). (D) Scaled residual fit between observed data and predicted model in (B). Compared to Fig 3C this suggests that, although the complexity of the species relatedness is not fully represented by the model, major gene flow events and their direction were probably captured. (E) Box plots of 30 runs of TreeMix (each started with a different seed for random number generation) likelihood at different numbers of allowed migration events; saturation starts after 3 additional migration edges. (F) Bar plot showing the number of times a particular directional migration event was inferred in 30 TreeMix runs with 5 migration events allowed. We show only the events which were inferred more than twice.

To further assess the complex demographic history of *Neobatrachus*, we performed TreeMix [51] modeling where species relationships are represented through a graph of ancestral populations (Fig 3A). The structure of the graph was inferred from allele-frequency data and Gaussian approximations of genetic drift such that the branch lengths in the graph are proportional to the amount of drift since populations split. We sequentially added up to 15 migration events, showing saturation of the model likelihood at five additional migration edges on average for 30 runs of Treemix, each with a different seed for random number generation (Fig 3E). We show an example of the inferred introgression events and the bifurcating graph for the model with five migration events for the run that resulted in the highest maximum likelihood (Fig 3A–3D). Inferred migration events (Fig 3B) indicate widespread directional introgression and interploidy gene flow between the polyploid species, however, only two introgression events had p-values lower than 0.05 in this particular run: from *N*. *sudellae* (4n) to *N*. *kunapalari* (4n) and from *N*. *sutor* (2n) to *N*. *kunapalari* (4n). Since there was some variability in inferred migration edges from run to run, to estimate the most frequently inferred migration events we summed the significant inferred migration edges among 30 TreeMix runs with five events allowed (Fig 3F). Migration events were found most frequently from *N*. *sudellae* (4n) to *N*. *kunapalari* (4n) (19 of 30 runs) and from *N*. *sutor* (2n) to *N*. *kunapalari* (4n) (12 of 30 runs). Interploidy introgression events were mostly asymmetric and from diploids to tetraploids, which corresponds with our ADMIXTURE cluster assignment results (Fig 1 and Fig 2). Inferred introgression events are broadly congruent with clusters of conflicting gene-tree topologies (S3 Fig). Each tetraploid *Neobatrachus* species (*N*. *aquilonius*, *N*. *kunapalari*, *N*. *sudellae*) is sister to a diploid species in the TreeMix graphs (Fig 3A and 3B, tips highlighted in bold) as well as in the species trees (Fig 1), which is consistent with previously suggested independent origins for the tetraploid species [19].

As an additional test for determining historical hybridizations, we estimated the network phylogeny of *Neobatrachus* using SNaQ [52] implemented in PhyloNetworks (version 0.11.0) [53]. We found that the scenario with two hybridization events allowed supported the data the best (see Methods, S9 Fig). The first inferred hybridization event suggests gene flow from the *N*. *aquilonius*, *N*. *fulvus*, and *N*. *sudellae* ancestral branch into *N*. *kunapalari* (minor hybrid edge γ = 0.343) (S9A Fig). The second inferred hybridization suggests gene flow from *N*. *sudellae* into *N*. *aquilonius* (minor hybrid edge γ = 0.192) (S9A Fig).

### Estimation of suitable distribution areas and demographic patterns

**Fig 4:**
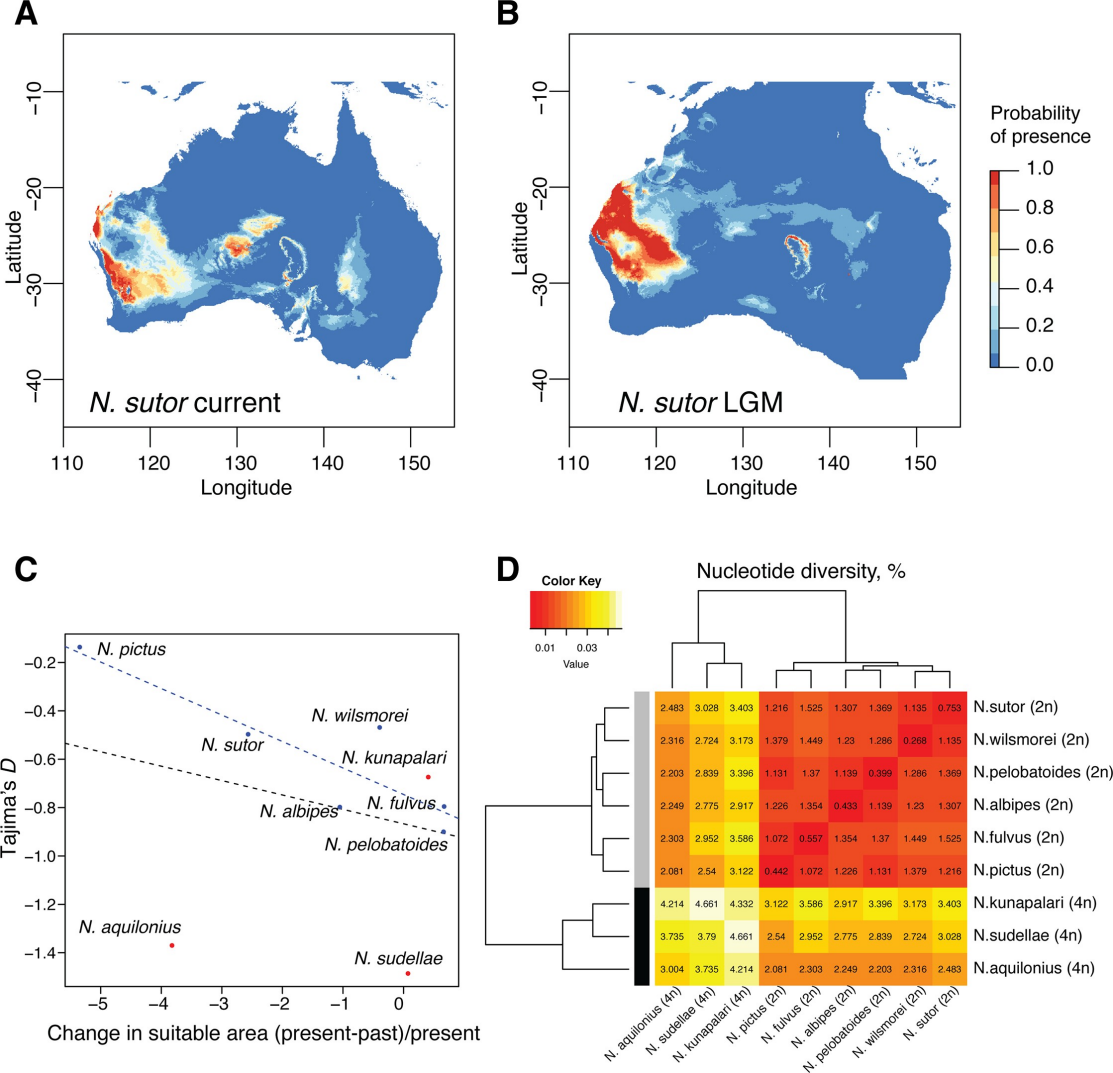
Diversity and differentiation of *Neobatrachus* species and geographical suitability estimates. (A) Example of the estimation of the suitable distribution area for *N*. *sutor*, based on occurrence data and current climate. (B) Example of the projection of the suitable distribution area for *N*. *sutor* based on the past climate at around 20Kya at LGM (last glacial maximum). Note that the scales in A and B are the same; Australian continent here is larger due to lower sea levels at LGM. (C) Scatter plot showing relative change of the predicted suitable area at the LGM and current conditions for each species as a function of Tajima’s D estimator. Diploid species show high correlation between Tajima’s *D* and distribution area change (blue line, Pearson’s correlation -0.88 (R^2^ = 0.72, p-value = 0.02); (D) Hierarchical clustering of *Neobatrachus* species based on mean nucleotide diversity within and between the species.

Tetraploid species have the highest nucleotide diversity among *Neobatrachus* species (Fig 4D and S2 Table), which is likely due to gene flow directed to tetraploid taxa and introgression between tetraploids of different origin. This is supported also by Fst distances (S10 Fig), where Fst distances between tetraploid species are the lowest, while Fst distances between tetraploids and diploid are larger, and Fst distances between the diploid lineages are the highest, suggesting stronger isolation.

To estimate the dynamics in population abundance over recent times, we measured Tajima’s *D*, a summary statistic that measures the lack or excess of rare alleles in a population compared to the neutral model. All of the *Neobatrachus* species have negative median values of Tajima’s *D*, which suggests that none of the species are experiencing dramatic population diversity decline (Fig 4C). We used the observed Tajima’s *D* values as a proxy for each species’ demographic patterns and compared them with estimated change in the suitable geographic area (Fig 4A–4C). In order to describe the ecological areas occupied by the different *Neobatrachus* species, as well as changes in those areas since the last glacial maximum (LGM) at around 20 Kya, we made use of occurrence data [54] (S11 Fig) and climate datasets [55]. We first performed a PCA of reduced bioclimatic variables concentrating on one of the highly correlated variables (r>0.85, Pearson correlation coefficient; *see* *Methods*) for individuals from the *Neobatrachus* occurrence data (S12 Fig). Using this substantially increased geographic sampling compared to our sequenced sample set, we could see moderate clustering of the individuals by species, which demonstrates that *Neobatrachus* species differ in their ecological (climatic) occupancies.

We then modelled suitable distribution areas for each species separately with MaxEnt [56], which applies machine learning maximum entropy modeling on the climate data at the geographical locations of the species occurrence data. Bioclimatic variables had different impacts on the model for each species (S13 Fig), however, appeared to be more similar for sympatric species (for example, *N*. *sutor* and *N*. *wilsmorei*) than for allopatric species (for example, *N*. *pictus* compared to any other diploid species). By projecting the models built on the current climate data on the past climate data (at the last glacial maximum, LGM) we could estimate the changes in the suitable habitat area since the LGM relative to the current suitable area for different species (Fig 4A–4C, S14 Fig and S15 Fig). We observed a correlation between the change in the suitable habitat area and median Tajima’s *D* for the diploid *Neobatrachus* species (Fig 4C). As shrinkage in suitable habitat areas increases for a given diploid species, Tajima’s *D* values also increase. This suggests that climate change since LGM to current days may have already had a negative effect on diploid *Neobatrachus’* genetic diversity via loss of suitable habitat even if populations are not obviously already getting smaller. Interestingly, tetraploid species appear to be the outliers to this trend, which we suggest may be due to their highly admixed genetic structure.

## Discussion

We have investigated the evolutionary history of the Australian burrowing frogs *Neobatrachus* genus and the population genomic consequences of genome duplication in a vertebrate model by generating and analysing nucleotide sequence data for 439 loci in 87 *Neobatrachus* individuals, covering the entire genus, including the three currently recognized tetraploids. The observation of non-bifurcating relationships between closely related species (Fig 1) is now common [57–67], and is understood to be caused by either rapid speciation, or shared variation between species due to incomplete lineage sorting (ILS) or gene flow. Population structure analysis revealed that each of the diploid species forms discrete clusters (Fig 1B and Fig 2), consistent with their status as phylogenetically distinct species. However, topology inconsistencies remained in the diploid only phylogenetic analysis (Fig 1C and S5 Fig). We show that random sets of individuals representing each species does not change the topology of a single locus and that inconsistency remains only between loci (S6 Fig). This suggests that diploid *Neobatrachus* species experienced rapid speciation and currently do not share variation between the species (no ILS or gene flow).

The tetraploids were assigned to a mixed set of clusters by population structure analysis, suggesting gene flow between each other and with the diploid species from overlapping geographical areas (Figs 1, 2 and S1 Fig). Comparing allele frequency distributions at the biallelic sites of the tetraploid individuals to the modelled expected distributions of autopolyploids with tetrasomic inheritance and allopolyploids with disomic inheritance, we rejected the hypothesis of the hybrid or allotetraploid origin of the two out of tree *Neobatrachus* tetraploid species–*N*. *sudellae* and *N*. *aquilonius* (S8 Fig). However, extensive gene flow between the autotetraploids of the different origin and from ‘non-parental’ diploids to the autotetraploids can lead to mixed inheritance, signs of which we see on *N*. *kunapalari* (4n) species (S8 Fig), for which we could not reject either of the inheritance modes.

Further extended analysis of the population structure and potential gene flow with TreeMix uncovered migration (gene flow) events from *N*. *sudellae* (4n) to *N*. *kunapalari* (4n) and from *N*. *sutor* (2n) to *N*. *aquilonius* (4n) (Fig 3). In several analyses, we inferred migration from *N*. *sudellae* (4n) to *N*. *aquilonius* (4n), reverse migration between *N*. *sutor* and *N*. *aquilonius*, migration from *N*. *albipes* (2n) to *N*. *kunapalari* (4n) and even *N*. *pelobatoides* (2n) to *N*. *albipes* (2n). The latter, if true, may be attributed to ancient migration events, since we do not see any evidence for recent mixing between the diploids (Figs 1 and 2, S6 Fig). As a complementary analysis we estimated a network phylogeny which recovered two hybridization events (S9 Fig): one between *N*. *sudellae* (4n) and *N*. *aquilonius* (4n), and a more ancient one between *N*. *kunapalari* (4n) and the ancestral branch of *N*. *aquilonius* (4n), *N*. *sudellae* (4n) and *N*. *pictus* (2n). This provided added support for gene flow between the tetraploid species. There are several possible scenarios of unidirectional gene flow from diploids to tetraploids which the TreeMix analysis suggested, for example, (1) through an unreduced gamete of a diploid crossing with a tetraploid or (2) through a triploid individual formed in a cross between diploid and a tetraploid, which could produce unreduced 3n gametes and backcross to a diploid. We describe these scenarios and potential mechanisms of unreduced gamete formation in more detail providing evidence based on literature [21, 25, 30, 31, 68–73], cytology and field observations in the S1 Text. Extensive gene flow between *Neobatrachus* species, especially between the tetraploid species, makes it difficult to estimate the true ancestral diploid population(s) for the tetraploids. Previously, it has been suggested that tetraploid *Neobatrachus* species might have independent origins [19]. Our results are well aligned with this suggestion and place tetraploid species in a polyphyletic arrangement on the species tree and on the TreeMix graphs, and suggest at least two independent origins of polyploidy: genetically closest diploid lineages to *N*. *aquilonius* and *N*. *sudellae* are *N*. *fulvus* and *N*. *pictus* respectively while the closest diploid lineage to *N*. *kunapalari* is *N*. *albipes*.

An important question that remains is what allows admixture between *Neobatrachus* tetraploids of potentially different origin and admixture of tetraploids with the local diploids, while the diploids seem to be currently isolated from each other? Similar to *Neobatrachus*, the tetraploid tree frogs *Hyla versicolor* of multiple origins *s*how high levels of interbreeding in overlapping geographical distribution, however the levels of divergence between the ancestral diploid species in this case are shallower [28]. Another example of a similar pattern was shown in plants, where polyploidy is more frequent: diploid *Arabidopsis lyrata* and *A*. *arenosa* do not hybridize, while tetraploidy seems to overcome the endosperm-based hybridization barrier enabling gene flow between the two species [74–76]. One hypothesis that can explain polyploidy mediated gene flow in *Neobatrachus* is that potentially incompatible loci in the diploids are masked in the tetraploids, however this requires a whole-genome sequencing analysis of introgression.

*Neobatrachus* species are widely distributed in Australia with tetraploid species occurring more in the central (drier) area compared to diploids, which is reflected in the principal component analysis of the climatic data for species occurrences (S12 Fig). Areas occupied by different *Neobatrachus* species differ only slightly in their environmental characteristics (S13 Fig). Worth mentioning is that climatic variables do not entirely describe ecological niches, which could differ in other characteristics such as timing of breeding and foraging, food source preference, or soil types etc. Nevertheless, ecological niche modelling based on climate data may provide additional insights into population dynamic trends. Here, we applied the MaxEnt modelling approach to the publicly available climate and occurrence data for all nine *Neobatrachus* species, comparing the present and past suitable geographical areas. Most of the *Neobatrachus* species showed substantial changes of the suitable areas comparing current and past presence probabilities (S14 Fig, S15 Fig). Interestingly, the estimated change in suitable habitat areas and population genetics estimator of demographic trends (Tajima’s *D*), obtained from independent datasets, were correlated (Fig 4C). Tetraploid species appear to be outliers from the general trend, probably due to their mixed population structure: in this case, emergence of rare alleles in the population due to migration events will affect Tajima’s *D* estimator. Overall, it appears that the species with greater shrinkage of suitable area since the last glacial maximum had less negative median Tajima’s *D* values, which suggests an ongoing shift from population expansion to population contraction.

## Conclusion and Outlook

*Neobatrachus* frogs represent a group of diploid and tetraploid species with a complex ancestry. By analysing sequence data of 439 targeted orthologous nuclear loci across the entire *Neobatrachus* genus we show rapid diversification of the diploid *Neobatrachus* species and multiple independent derivations of the tetraploid species. This work also revealed that the diploid *Neobatrachus* species are currently isolated, when autotetraploids of a different origin are able to hybridise with each other and with the local diploids in a unidirectional manner with gene flow from the diploids to the tetraploids. Tetraploid *Neobatrachus* species are able to occupy harsher environments and are distributed more widely across Australia. We suggest that polyploidy-mediated gene flow and hybridisation promote the adaptive advantage of the tetraploids in the face of climate change. These results, revealing gene flow between tetraploids and asymmetric inter-ploidy gene flow, pose a number of important questions concerning the evolution of sexual polyploid animals. Whole-genome sequencing data for *Neobatrachus* species would not only help to refine the population structure and introgressive mixing in the genus, but also provide information on potential adaptive values of the introgressed regions. One could hypothesize that a wide and potentially rapid spread of the tetraploids into new territories was facilitated by introgression from the locally adapted diploids [77], and that more detailed sampling of the tetraploids from parts of their distribution ranges remote from sympatry with diploids may reveal evidence of early versus ongoing gene flow. Moreover, population-level genomic resequencing of multiple diploid and tetraploid sister species could provide insight into the unique biology of autotetraploid sexual animals and effects of the tetraploidization on their evolution. The results on the changing suitable habitat areas for the *Neobatrachus* species highlight the importance of continuous observation of their population dynamics. Monitoring the current status of biodiversity through collection of species occurrence data and population genetic data allows the prediction of population dynamics and hopefully timely responses in conservation efforts in the face of rapidly changing environments [78, 79]. Emerging methods of public engagement to collect occurrence and other data (video and audio; www.frogid.net.au, [80, 81]) have potential to provide essential information on the state of frog species.

## Methods

### Anchored Hybrid Enrichment (AHE) phylogenomics

All the samples examined were obtained from the Australian Biological Tissue Collection at the South Australian Museum. Details of all samples examined are presented in the S1 Table. We collected AHE data at Florida State University’s Center for Anchored Phylogenomics (www.anchoredphylogeny.com), following the methods described in Lemmon et al. [41] and Prum et al. [82]. Briefly, after quantifying the extracted DNA using Qubit, we sonicated the DNA to a size range of 150-500bp using a Covaris Ultrasonicator. We then prepared indexed libraries using a Beckman Coulter FXp liquid-handling robot. After library QC using Qubit, we pooled the libraries in groups of 16 and enriched the library pools using an hybrid enrichment kit developed for use in Anurans [42, 43]. Finally, we sequenced the enriched library pools on two lanes of an Illumina 2500 sequencer with a PE150 protocol at the Translational Laboratory at Florida State University.

Following sequencing, we quality filtered the reads using the Casava high-chastity filter, then demultiplexed the reads using the 8bp indexes with no mismatches tolerated. To increase read length and correct for sequencing errors, we merged read pairs that overlapped by at least 18bp using the method of Rokyta et al. [83]. This process also removed sequencing adapters. We then performed a quasi ‘de novo’ assembly of the reads following Hamilton et al. [84], with *Pseudacris nigrita*, and *Gastrophryne carolinensis* as references. In order to reduce the potential effects of low level sample contamination, we retained only the assembly clusters containing more than 61 reads. In order to produce phased haplotypes from the assembly clusters, we applied the Bayesian approach developed by Pyron et al. [85], in which reads overlapping polymorphic sites are used to identify the likely phase of allelic variants within each locus. Because this approach was developed to accommodate any ploidy level, we were able to isolate two or four haplotypes for diploid and tetraploid individuals, respectively. We determined orthology for each locus using a neighbor-joining approach based on pairwise sequence distances, as described in Hamilton et al. [84]. We aligned homologous haplotypes using MAFFT v7.023b [86], then auto-trimmed/masked the alignments following the approach of Hamilton et al. [84], but with *MINGOODSITES* = 12, *MINPROPSAME* = 0.3, and *MISSINGALLOWED* = 48. Final alignments were visually inspected in Geneious R9 (Biomatters Ltd., [87]) to ensure that gappy regions were removed and misaligned sequences were masked.

### Misidentifications in the dataset

Both phylogenetic and admixture assignments suggested that several individuals had been misidentified in the field, which is expected for morphologically similar species and in particular for some of the diploid-tetraploid species pairs, e.g. *N*. *fulvus* and *N*. *aquilonius* [20]. Field sampling can be accompanied by a certain level of honest mistakes in species identification, especially for sympatric species. However, a high level of incompletely sorted polymorphisms in recently split lineages or recent hybridization events could also result in uncertain positioning of an individual. We carefully curated the dataset and made a decision to rename some of the misidentified samples or completely exclude them from the analyses based on the amount of the missing data in the assembly, ploidy estimations from the sequencing data, and on the clear placement in a different clade. Below we describe our workflow for manual curation of the dataset to exclude or rename uncertain individuals without compromising too much on the potentially real shared variation.

The multiple sequence alignment resulting from the AHE workflow contains different amounts of informative sequence and gaps for each individual. First, we calculated the informative sequence fraction (no gaps) for each individual compared to the multiple sequence alignment length and applied a threshold of at least 0.2 of informative fraction for each individual to qualify for the subsequent analysis. Based on these criteria we excluded 6 samples (S1 Table).

Second, we estimated the ploidy of each sample using the nQuire software [88] on the next generation sequencing data mapped to one of the outgroup species *Heleioporus australiacus* (I5549) AHE assembly as a reference. As a preparation step for nQuire, we mapped reads to the reference using the BWA-MEM algorithm from BWA [89] (version 0.7.17), used Samtools [90] (version 1.6) to sort and index the mapping and removed potential duplicates from the PCR amplification step of library preparation with picard-tools (http://broadinstitute.github.io/picard/). We used the denoised input of base frequencies generated with default parameters for the Gaussian Mixture Model utilized in nQuire to estimate ploidy levels on the basis of frequency distributions at biallelic sites. The resulted estimations can be found in S1 Table. We excluded 5 samples placed in a different clade compared to the rest of the samples in the corresponding lineage, where ploidy estimation confirmed their misidentification. Finally, we renamed 2 samples to a different species name with which it clustered, in cases when initial ploidy and estimated ploidy corresponded to each other (S1 Table).

We have also excluded from further analysis sample I5442, initially identified as *N*. *kunapalari*, which was estimated to be a triploid and showed high levels of admixture between a diploid *N*. *wilsmorei* and potentially *N*. *kunapalari* or *N*. *sudellae*. In fact, triploid individuals are known in natural populations of *Neobatrachus* [22, 24, 25, 91, 92]^,^ (S1 Text), and could provide an explanation for the gene flow between species of different ploidy through a “triploid bridge”. A 3n individual formed in a cross between 2n and 4n individuals could produce a haploid, diploid or triploid gametes. Diploid gametes can cross to a tetraploid and produce 4n individuals that can backcross into the sympatric 4n population [71, 93, 94].

### Phylogenetic analysis

To generate a molecular species tree, we started by reconstructing individual genealogies for each of the 439 recovered loci. We analyzed two datasets, of which the first included all samples (except triploids discussed in the Results section), while the second was trimmed down to just two individuals per species. Results from the full sampling can be found in the *Supplementary Material* (S2 Fig), and the finer sampling in the main text (Fig 1A). We used RaxML [46] to simultaneously search for the best tree and apply 100 rapid bootstraps, implementing the GTRGAMMA model of nucleotide evolution for each locus. In generating species trees, coalescent methods have been shown to be more accurate than concatenation in cases of extensive incomplete lineage sorting, so we used the shortcut coalescent method ASTRAL III. Shortcut coalescent methods like ASTRAL take individual gene trees as input, and are much more computationally efficient than full coalescent analyses. We used our RAxML-generated gene trees as input for ASTRAL, allowing us to make use of all our molecular data.

To address gene-tree incongruence in a diploid-only phylogeny and investigate possible conflicting signals in our data as a result of (1) rapid radiation or (2) introgression and/or incomplete lineage sorting (ILS), we took a two-fold approach. We started by randomly selecting loci with complete species-level sampling for *Neobatrachus*, and visualized these gene trees with a single representative per species, varying the sampled individuals among plots to visualize the consistency of interspecific relationships (S6 Fig). Following this exercise we used multidimensional scaling (MDS) to approximate the relative distances between gene tree topologies [95]. To prepare the data, we again trimmed down gene trees to one sample per species of *Neobatrachus*, and discarded loci missing any taxa, leaving us with 361 loci. We started by simply visualizing gene-tree incongruence overlaying the topologies of all 361 loci in DensiTree (Fig 1, S5 Fig). We then calculated the pairwise distances between all gene trees using the Robinson-Foulds metric, in the R package APE [96]. We projected the tree distances into two and three dimensions (representing tree topology space) using MDS, as visualizing and interpreting more dimensions is difficult. To test if gene trees are uniformly distributed throughout tree space, or clustered, we used the partitioning around the medoids algorithm as implemented in the R package CLUSTER [97]. We chose the optimum number of clusters (*k*), using the gap statistic, calculated for each *k* = 1–10. Clusters of gene trees represent similar topologies, and so we then summarized each cluster using ASTRAL, to identify consistent differences in topology.

The inclusion of tetraploid taxa with admixed genetic material has the potential to negatively influence bifurcating species tree inference. To address this, we removed tetraploid taxa from all alignments to jointly estimate a species tree and divergence dates for diploid *Neobatrachus* species using StarBEAST2 [98]. We chose two individuals per diploid *Neobatrachus* and *Heleioporus* species, as well as five microhylid outgroup taxa (*Cophixalus cheesmanae*, *Liophryne rhododactyla*, *Microhyla achatina*, *Kalophrynus interlineatus*) for calibration purposes. Full coalescent species tree inference can be computationally prohibitive with large data matrices, and so we chose 25 loci selected for sampling completeness, locus length, and number of variable sites informed by AMAS [99] (S7 Fig). We used a single strict molecular clock linked across the 25 partitions, independent GTR site models, and ran four independent chains for 1x10^9^ generations, sampled each 5x10^5^ generations. Because no valuable fossil information for *Neobatrachus* is available, we used two secondary calibrations from the most extensive anuran time-tree to date [48]. The first on the root of the tree (split between Microhylidae and Myobatrachidae; normal distribution, mean = 128, sigma = 4), and the second between *Neobatrachus* and *Heleioporus* (normal distribution, mean = 32.5, sigma = 4). Each run was inspected for stationarity with TRACER [100], and summarized to a maximum clade credibility tree with mean heights to check for consistency in topology and divergence times.

While bootstrapping and posterior probabilities are commonly used to investigate topological confidence, individual gene trees arguably provide a better estimate of concordance in branching events found in the species tree. We estimated gene concordance factors (GCF) in IQTREE [101], and plotted them along the species tree to visualize support for branching patterns in the *Neobatrachus* species tree (Fig 1C and S5 Fig).

### Population structure

Maximum likelihoods of individual ancestries were estimated with ADMIXTURE [47] for 66,789 biallelic sites combining all 439 loci and allowing for maximum 20 out of 87 *Neobatrachus* individuals (excluding the outgroup species) to have missing data at each site. In order to include tetraploid samples in ancestry assignment we randomly chose two alleles for each site. We also applied minor allele frequency threshold of two percent. Ancestral population assignment showed three local minima of cross-validation errors at K equals 3, 7 and 9 (S4 Fig), with K = 7 being the lowest, which we chose for the subsequent analysis as the optimal solution.

### Inheritance mode

In order to check the inheritance mode of the three tetraploid *Neobatrachus* species we compared allele frequencies distributions at biallelic sites within each individual for each species with modeled expected distributions for auto and allo tetraploids. Autotetraploids were modelled combining bam files within each diploid species from the mapping described above; allotetraploids were modelled combining bam files between each diploid species (S8A Fig). Base frequencies distributions of biallelic sites were produced using the denoised algorithm from nQuire [88] for each individual separately. The base frequencies for each individual have a continuous rather than discrete (AAAB 0.25, AABB 0.5, ABBB 0.75) distribution since they are calculated from read counts at a biallelic site. As it was previously shown [49, 50] allotetraploids with disomic inheritance mode are expected to have an excess of intermediate frequency alleles, which is supported by our models as well (S8A Fig). Performing Wilcoxon tests for the ratios between intermediate (40–60%) and rare (<30%) allele frequencies we rejected allotetraploid origins for *N*. *sudellae* and *N*. *aquilonius*. *N*. *kunapalari* showed intermediate distributions, suggesting mixed chromosomal inheritance. A mixed chromosomal inheritance pattern can be explained under several scenarios including being newly formed allopolyploid hybrids of close relation, the presence of continued gene-flow with diploids or other autopolyploid species, or the process of diploidization in an autopolyploid with tetrasomic inheritance [102]. Other analyses in this study suggest extensive gene flow between *N kunapalari* and diploids as well as tetraploids (Fig 2, Fig 3B and 3F). While we cannot rule out the possibility that *N*. *kunapalari* was initially formed from the hybridization of two closely related lineages, we believe extensive gene flow and the older lineage age of *N*. *kunapalari* is a sufficient and more likely cause for its elevated intermediate allele frequencies when compared to the other tetraploids.

### Introgression inference

The graphs representing ancestral bifurcations and migration events were produced using Treemix V.1.12 [51]. Input data contained 5092 biallelic sites called at 439 loci among 9 *Neobatrachus* species with at least 20% of the data to be present at each species at each site. Position of the root was set to *N*. *pelobatoides* as the nuclear species tree suggested (Fig 1A, black). To account for linkage disequilibrium we grouped SNPs in windows of size 10 using -k flag. We also generated bootstrap replicates using -bootstrap flag and subsequently allowed up to 15 migration events with flag -m. We ran TreeMix software with 30 different random number generated seeds. For graph and residuals visualisation we used R script plotting_funcs.R from the Treemix package.

The network phylogeny of *Neobatrachus* was estimated using SNaQ [52] implemented in PhyloNetworks (version 0.11.0) [53]. We used a single individual for each species, and estimated gene tree phylogenies in RAxML [46], as described in the phylogenetic analysis section above, using *Heleioporus australiacus* as the outgroup taxon, but pruned *Heleioporus* from all trees for our PhyloNetworks analysis so as not to infer spurious ingroup-outgroup hybridizations as can occur in network analyses. We used a species tree estimated by ASTRAL as the starting tree for our analysis, allowing for between 0 and 10 hybridizations. We conducted 10 replicates for each hybridization value. Final trees were re-rooted with *Neobatrachus pelobatoides* as the root to match the network tree with the major tree. Pseudolikelihood values reported from PhyloNetworks demonstrate a sharp increase in support when allowing 1 (-Ploglik = 55.06) hybridization event as opposed to 0 (-Ploglik = 188.17). We also found a considerable change when allowing for 2 hybridization events (-Ploglik = 48.22). With more than two hybridizations allowed, however, PhyloNetworks only recovered two hybridization events or additional hybridizations resulted in suboptimal pseudolikelihoods (S9 Fig).

### Summary statistics and demographic tendencies

We calculated summary statistics (Fig 4A–4C) with the R package “PopGenome” [103] for all the loci with more than 100 aligned sites (filtering for only non-variable and biallelic sites and filtering out sites with more than two alleles) separately for each species for within-species statistics (nucleotide diversity, Tajima’s *D*, S2 Table) and in a pairwise mode for between-species statistics (Fst).

### Species distribution modelling

Bioclimatic variables were obtained from worldclim project [55] with 2.5 minutes resolution for reconstructed climate data at Last Glacial Maximum around 20Kya, averaged conditions across 1960–1990 and the most recent available conditions averaged across 1970–2000. Software DIVA-GIS 7.5 was used to trim the data to area longitude from 110 to 155 and latitude from -40 to -9. Bioclimatic variables were excluded if they were highly correlated (r>0.85, Pearson correlation coefficient) in all 3 climatic data sets, leaving for further analysis 6 bioclimatic variables in total: BIO9 = Mean Temperature of Driest Quarter, BIO10 = Mean Temperature of Warmest Quarter, BIO12 = Annual Precipitation, BIO17 = Precipitation of Driest Quarter, BIO18 = Precipitation of Warmest Quarter, BIO19 = Precipitation of Coldest Quarter.

To model the species suitable area we used software MaxEnt (v. 3.4.1.), which predicts species distribution from climate data using the species occurrences employing a machine learning technique called maximum entropy modeling [56]. Here we used *Neobatrachus* species occurrence data from amphibiaweb.org [54], which includes 189 entries for *N*. *albipes*, 282 for *N*. *aquilonius*, 87 for *N*. *fulvus*, 588 for *N*. *kunapalari*, 802 for *N*. *pelobatoides*, 699 for *N*. *pictus*, 707 for *N*. *sudellae*, 639 for *N*. *sutor* and 282 for *N*. *wilsmorei* (S11 Fig). We used 75% of occurrence points for each species for model training and 25% for model testing with 1,000,000 background points and 10 replicates. We have trained the model on bioclimatic variables (reduced to 6 in total, described earlier) averaged across conditions 1960–1990; and then projected that model to the same set of environmental variables from the Last Glacial Maximum. The average test AUC (area under the Receiving Operator Curve) for the replicate runs for all the species was more than 0.9 (S3 Table), indicating a high performance of the models. In order to estimate which bioclimatic variable is the most important in the models we performed a jackknife test, where model performance was estimated without a particular variable and only with this particular variable in turn (S13 Fig).

We used cloglog output format of MaxEnt, which gives an estimate between 0 and 1 of probability of presence of the species in the area. In order to determine the relative change of the suitable area we used the point-wise mean values from the 10 replicates for model predictions on current and past climate (S14 Fig and S15 Fig). We extracted the suitable area for both climate conditions with R library ‘raster’ [104] with 0.8 presence probability threshold and estimated change in the suitable area relative to the current suitable area as (current-past)/current.

## Supporting information

S1 Text Summary of cytogenetic observations and mechanisms for unidirectional introgression. (DOCX)

S1 Table Sample information, BioSample IDs, metadata, ploidy inference and filtering. (XLSX)

S2 Table Summary statistics for each of the species calculated with R package “PopGenome”. (XLSX)

S3 Table The average test AUC (area under the Receiving Operator Curve) for the replicate runs for all the species in MaxEnt modeling for predicting species distribution from climate data at the species occurrences. (XLSX)

S4 Table Instances of polyploid *Neobatrachus*. (XLSX)

S1 Fig Species tree and admixture results for optimal clustering at K equals 3, 7 and 9 (see S4 Fig. for optimal number of clusters). Vertical colored bars to the left of the tips of the tree correspond to our final species assignments (S1 Table); colors of the bars are species-specific and correspond to the branch colors from Fig 1A; filtered out samples are marked with black bars.
(TIF)

S2 Fig Nuclear species tree as inferred using ASTRAL, all nuclear loci, and complete taxon sampling. Figure extends across four parts (A, B, C, D) and is color coded by species identity.
(TIF)

S3 Fig Two dimensional representations of MDS gene tree space, colored by optimal clustering scheme for two dimensions (k = 2) and three dimensions (k = 4), and their associated topologies inferred using ASTRAL. Each point represents a single gene tree, colored clusters match colored trees displayed to the right. Nodes at values indicate bootstrap support.
(TIF)

S4 Fig Cross-validation plot showing three local optimal solutions for ADMIXTURE clustering at K equals 3, 7 and 9. (TIF)

S5 Fig (A) Gene trees, colored by clade, for 361 nuclear loci based on 2 individuals per species show considerable incongruence and differ from the species trees (bold black topology). (B) Gene trees for diploid individuals only also show considerable incongruence and differ from the species trees (bold black topology). (C,D) Species tree colored by topological consistency as measured by gene concordance factors—gCF%, the percentage of loci which decisively favor a given bipartition. Warmer colors indicate high discordance, cooler colors indicate strong concordance.
(TIF)

S6 Fig Genealogies for six randomly sampled nuclear loci (y-axis) with different diploid individuals chosen as representatives for each species (different sample sets, x-axis) are consistent with each other. Genealogical conflict remains only among loci. This supports a scenario of rapid speciation of the diploid species without secondary contact or persistent incomplete lineage sorting.
(TIF)

S7 Fig Sequenced loci statistics on alignment length and number of variable sites inferred by AMAS (11). (TIF)

S8 Fig Distribution of allele frequencies of biallelic sites in *Neobatrachus* tetraploids supports tetrasomic inheritance mode in *N*. *sudellae* and *N*. *aquilonius* and mixed inheritance mode in *N*. *kunapalari*. (A) Pairwise combination of individuals within the diploid species model the expected allele frequencies in autotetraploids with tetrasomic inheritance (blue line), when pairwise combination of individuals between the diploid *Neobatrachus* species model the expected distribution for allotetraploids with disomic inheritance mode (purple line). Modeled allotetraploids show excess of intermediate allele frequencies compared to autotetraploids. Gray area shows 95% confidence interval. (B) Comparing the ratio between intermediate (40–60%) and rare (<30%) allele frequencies we reject allotetraploid origin for *N*. *sudellae* and *N*. *aquilonius*, when *N*. *kunapalari* shows intermediate distribution, suggesting mixed inheritance. Comparisons performed with Wilcoxon tests adjusted for multiple testing.
(TIF)

S9 Fig SnaQ analysis. A. The optimum phylogenetic network includes two hybridization events. B. Network score has the best support at minumum 2 hybridization events, additional allowed hybridizations do not increase the network score.
(TIF)

S10 Fig Heatmap and hierarchical clustering of the *Neobatrachus* lineages based on the distance matrix from pairwise median Fst values. Tetraploid species (*N*. *sudellae*, *N*. *aquilonius* and *N*. *kunapalari*; highlighted with black left bar) cluster together and are characterised by the lowest Fst values between each other. This, together with low Fst values between tetraploid and diploid lineages, can probably be explained by the gene flow within the tetraploids and between the diploids and the tetraploids. Diploid lineages (highlighted with grey left bar) appear to be more isolated from each other compared to tetraploids, which is in agreement with ADMIXTURE assignment results and TreeMix estimations of possible migration events.
(TIF)

S11 Fig Occurrence data locations registered at the AmphibiaWeb database for Neobatrachus species: A—tetraploids, B—diploids. (TIF)

S12 Fig PCA analysis of bioclimatic variables for *Neobatrachus* entries in the occurrence AmphibiaWeb database. A) Barplot showing the percentage of variances explained by each principal component. The first three principal components are labeled with the top three contributions of variables. BIO10 = Mean Temperature of Warmest Quarter, BIO12 = Annual Precipitation, BIO17 = Precipitation of Driest Quarter, BIO18 = Precipitation of Warmest Quarter, BIO19 = Precipitation of Coldest Quarter. B-D) Pairwise combinations of the first three principal components, where individuals with a similar profile of bioclimatic data are grouped together. Points represent each individual and colored according to the species assignment, ellipses represent 95% confidence area.
(TIF)

S13 Fig The results of the jackknife test of variable importance for models on each species. BIO19 (Precipitation of Coldest Quarter) was the most informative variable for the models of *N*. *pelobatoides* and *N*. *albipes* distributions; BIO18 (Precipitation of Warmest Quarter) was the most informative variable for the models of *N*. *wilsmorei*, *N*. *sutor* and *N*. *kunapalari*; BIO17 (Precipitation of Driest Quarter) was the most informative variable for the model of *N*. *fulvus*; BIO10 (Mean Temperature of Warmest Quarter) for *N*. *pictus*; and BIO9 (Mean Temperature of Driest Quarter) for *N*. *sudellae* and *N*. *aquilonius*.
(TIF)

S14 Fig The point-wise mean of the 10 models for each of the diploid species build on environmental layers from the current climate data and applied to the environmental layers from the Last Glacial Maximum climate data. (TIF)

S15 Fig The point-wise mean of the 10 models for each of the tetraploid species build on environmental layers from the current climate data and applied to the environmental layers from the Last Glacial Maximum climate data. (TIF)

S16 Fig Karyotypes of *Neobatrachus*. A) *N*. *sutor* [2n], B) *N*. *pictus* x *N*. *sudellae* triploid [3n] hybrid from Moyston, east of the Grampians, Victoria, C) *N*. *fulvus* x *N*. *sutor* triploid [3n] hybrid from Learmonth, Western Australia, D) *N*. *sudellae* [4n], E) tetraploid x tetraploid hybrid from north of Menzies, Western Australia, F) *N*. *pictus* x *N*. *sudellae* pentaploid [5n] hybrid from Moyston, east of the Grampians, Victoria. Arrowheads indicate nucleolar organiser regions (NORs).
(TIF)
