# Supplementary material for: Polyploidy breaks speciation barriers in Australian burrowing frogs Neobatrachus
Source: PLoS Genet. 2020 May 11;16(5):e1008769. doi: 10.1371/journal.pgen.1008769 (PMC7259803; doi:10.1371/journal.pgen.1008769)
Supplement: S1 Text — (DOCX) [file pgen.1008769.s001.docx]

**S1 Text. Summary of cytogenetic observations and mechanisms for unidirectional introgression**

Unidirectional gene flow from diploid to tetraploids could be envisioned through a couple of scenarios. Firstly, a diploid individual could produce an unreduced gamete in a cross with a tetraploid forming a tetraploid that could backcross with the sympatric tetraploid species. While cytological evidence of the production of unreduced gametes in diploids is available in the form of triploid hybrids between diploid *Neobatrachus* species (S16C Fig), we presently do not have direct evidence of this first scenario for inter-ploidy gene flow in *Neobatrachus* (S4 Table) (1-5).

Secondly, a diploid individual could cross with a tetraploid and form a triploid. A triploid producing an unreduced gamete (3n) in a cross with a diploid (normal 1n gamete) would produce a tetraploid that could backcross to a tetraploid individual producing introgression from the diploid into the tetraploid gene pool, i.e. the so-called “triploid bridge” (6). We have cytological and molecular genetic evidence of naturally occurring triploid *Neobatrachus* and direct cytological evidence of triploid *Neobatrachus* producing 3n gametes. For example, triploid *pictus* (2n) x *sudellae* (4n) hybrids (S16B Fig) have been observed at hybrid zones between *N. pictus* and *N. sudellae* on the Eyre Peninsula, South Australia and at Moyston east of the Grampians, Victoria (Mahony 1986, unpublished data) (Table S3). Another evidence of possible triploid formation comes from diploid x tetraploid species combination from viable laboratory crosses of *N. sutor* (2n) x *N. kunapalari* (4n) produced by Main (1962) (Table S3). Here we also provide a molecular genetic evidence of triploid formation through hybridisation through triploidy inference resulting from a cross between *wilsmorei* (2n) x *kunapalari* (4n) or *sudellae* (4n) – sample number I5442 (WAM R119302) from Yalgoo, WA (see S1 Table on ID corrections), however this sample was excluded from the further analysis. Moreover, the evidence that triploids can produce 3n gametes comes from the presence of a pentaploid *pictus* x *sudellae* hybrid (S16F Fig) at a *pictus* (2n) x *sudellae* (4n) hybrid zone at Moyston, east of the Grampians, Victoria where triploid *pictus* x *sudellae* hybrids were observed also (Mahony 1986, unpublished data) (Table S3). Although there is no direct evidence that a triploid producing an unreduced gamete (3n) in a cross with a diploid (1n gamete) would produce a tetraploid which could backcross to a 4n individual producing introgression from the diploid into the tetraploid gene pool, i.e the so-called “triploid bridge”, the provided circumstantial evidence suggest this as a possible mechanism. Moreover, in another polyploid frog complex, *Odontophrynus*, triploids produced in laboratory crosses formed haploid, diploid and triploid gametes (1), which opens the possibility of triploids crossing with diploids or tetraploids to produce tetraploids that could backcross into the sympatric tetraploid gene pool.

Selective mating scenarios based on call characteristics could increase the likelihood of the backcross matings with tetraploids (2). It is also worth mentioning that *Neobatrachus* species are explosive breeders and require very heavy rains (>25 mm in a rainfall) to promote emergence. In such events, sympatric species, for example *N. kunapalari*, *N. pelobatoides* and *N. albides* can all be breeding in the same ponds or pools (unpublished observation). A recent study on *Bufo japonica*, also an explosive breeder, showed that multiple paternity occurred commonly in that species in ponds with high densities of amplexed pairs but not in ponds with low density populations (7).  The inferred cause was stray sperm in the pond from other amplexed pairs or possibly unpaired males.

A mechanism for unreduced gamete formation, i.e. temperature stress, has been identified experimentally (8, 9 ). Similar brief stress conditions during zygote development before the first cell division could block the first division and form a tetraploid that could cross into a sympatric tetraploid gene pool (3, 10). Cold or heat shocks are possible for *Neobatrachus* egg clutches at numerous locations across southern Australia that experience temperature extremes of the order of −5 °C to 48 °C. For instance Roberts and Edwards (2018) (4) note that the minimum maximum temperature range for Hyden WA as −5.6 °C to 48.6 °C. Breeding by more southerly *Neobatrachus* tends to follow the onset of autumn and winter rains, typical of the Mediterranean climate in this region. Exposure to freezing or near freezing conditions in shallow egg deposition sites following cold fronts associated with rain fall events is frequently possible. *Neobatrachus* in the arid zone breed in association with heavy summer rains, which may be associated with heat wave conditions.

1. Becak ML, Becak W. Further studies on polyploid amphibians (Ceratophrydidae). 3. Meiotic aspects of the interspecific triploid hybrid: Odontophrynus cultripes (2n=22) x O. americanus (4n=44). Chromosoma. 1970;31(4):377-85.

2. Main AR. Comparisons of breeding biology and isolating mechanisms in Western Australian frogs. (ed.) GWL, editor. Melbourne, Victoria, Australia: Melbourne Univ. Press 1962. 370-9 p.

3. Nishioka M, Ueda H. Studies on polyploidy in Japanese frogs. Sci Rep Lab Amphibian Biol Hiroshima Univ. 1983;6:207–52.

4. Roberts JD, Edwards D. The Evolution, Physiology and Ecology of the Australian Arid-Zone Frog Fauna. On the Ecology of Australia’s Arid Zone2018. p. 149-80.

5. Roberts JD, Mahony M, Kendrick P, Majors CM. A new species of burrowing frog, Neobatrachus (Anura:Myobatrachidae), from the eastern wheatbelt of Western Australia. Records of the Western Australian Museum. 1991;15:23-32.

6. Bogart JP, Bi K. Genetic and genomic interactions of animals with different ploidy levels. Cytogenet Genome Res. 2013;140(2-4):117-36.

7. Shimada M, Hase K. Female polyandry and size-assortative mating in isolated local populations of the Japanese common toad Bufo japonicus. Biological Journal of the Linnean Society. 2014;113(1):236-42.

8. Mason AS, Pires JC. Unreduced gametes: meiotic mishap or evolutionary mechanism? Trends Genet. 2015;31(1):5-10.

9. Pandian TJK, R. Ploidy induction and sex control in fish. Hydrobiologia. 1998;384(1-3):167-243.

10. Geach TJ, Stemple DL, Zimmerman LB. Genetic analysis of Xenopus tropicalis. Methods Mol Biol. 2012;917:69-110.
