# Supplementary figures and images for: Polyploidy breaks speciation barriers in Australian burrowing frogs Neobatrachus
Source: PLoS Genet. 2020 May 11;16(5):e1008769. doi: 10.1371/journal.pgen.1008769 (PMC7259803; doi:10.1371/journal.pgen.1008769)

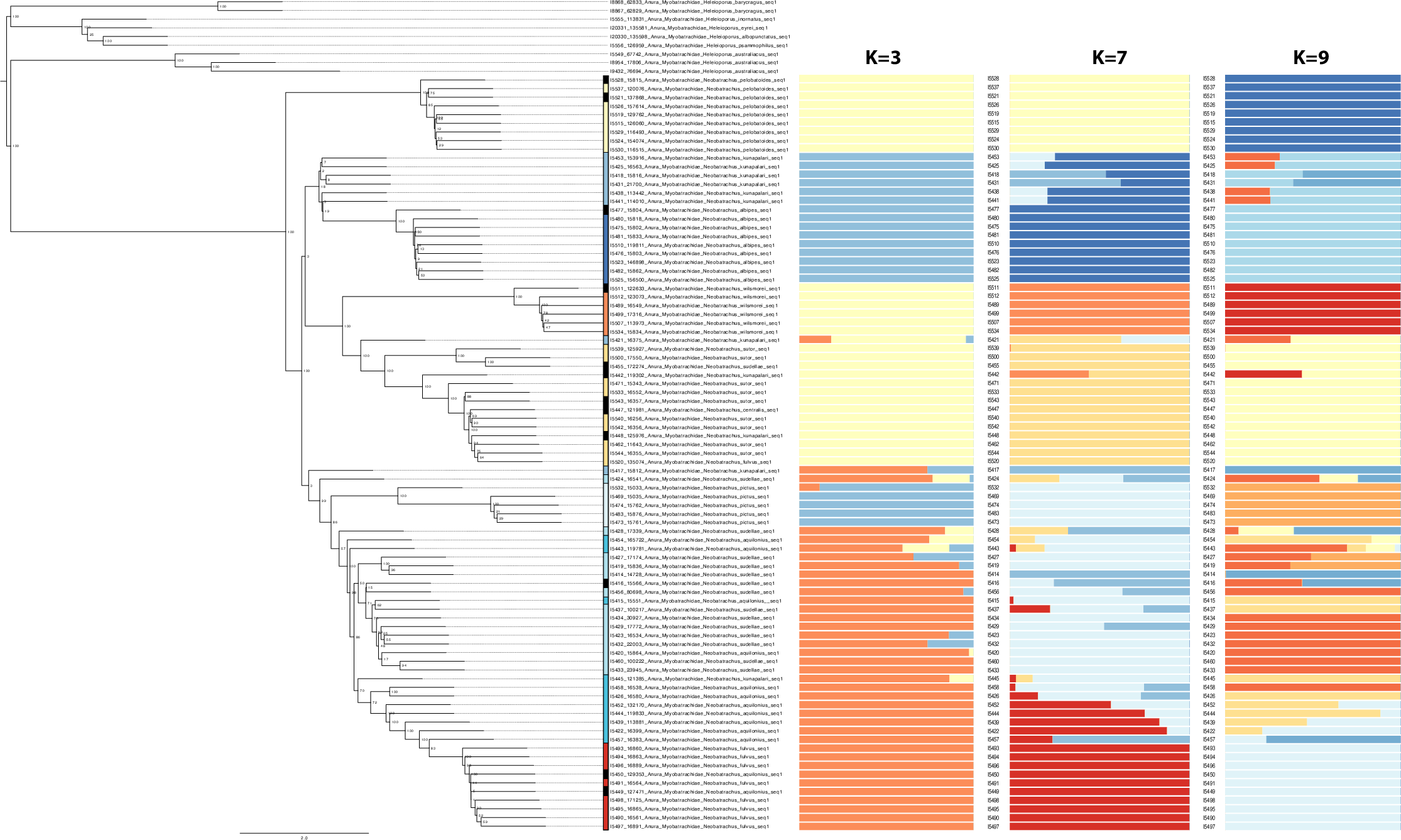

Supplement: S1 Fig — Vertical colored bars to the left of the tips of the tree correspond to our final species assignments (S1 Table); colors of the bars are species-specific and correspond to the branch colors from Fig 1A; filtered out samples are marked with black bars. (TIF) [file pgen.1008769.s006.tif]

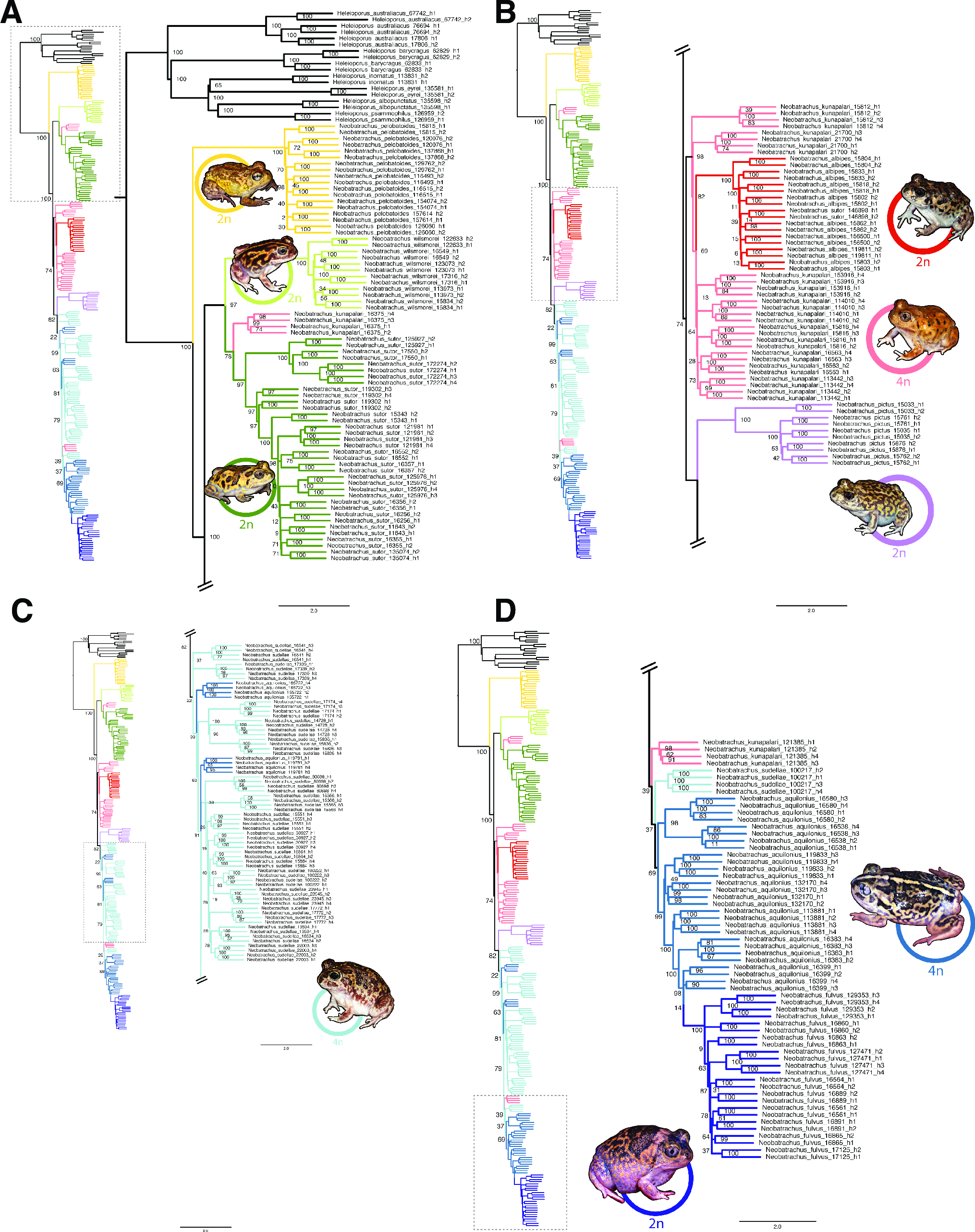

Supplement: S2 Fig — Figure extends across four parts (A, B, C, D) and is color coded by species identity. (TIF) [file pgen.1008769.s007.tif]

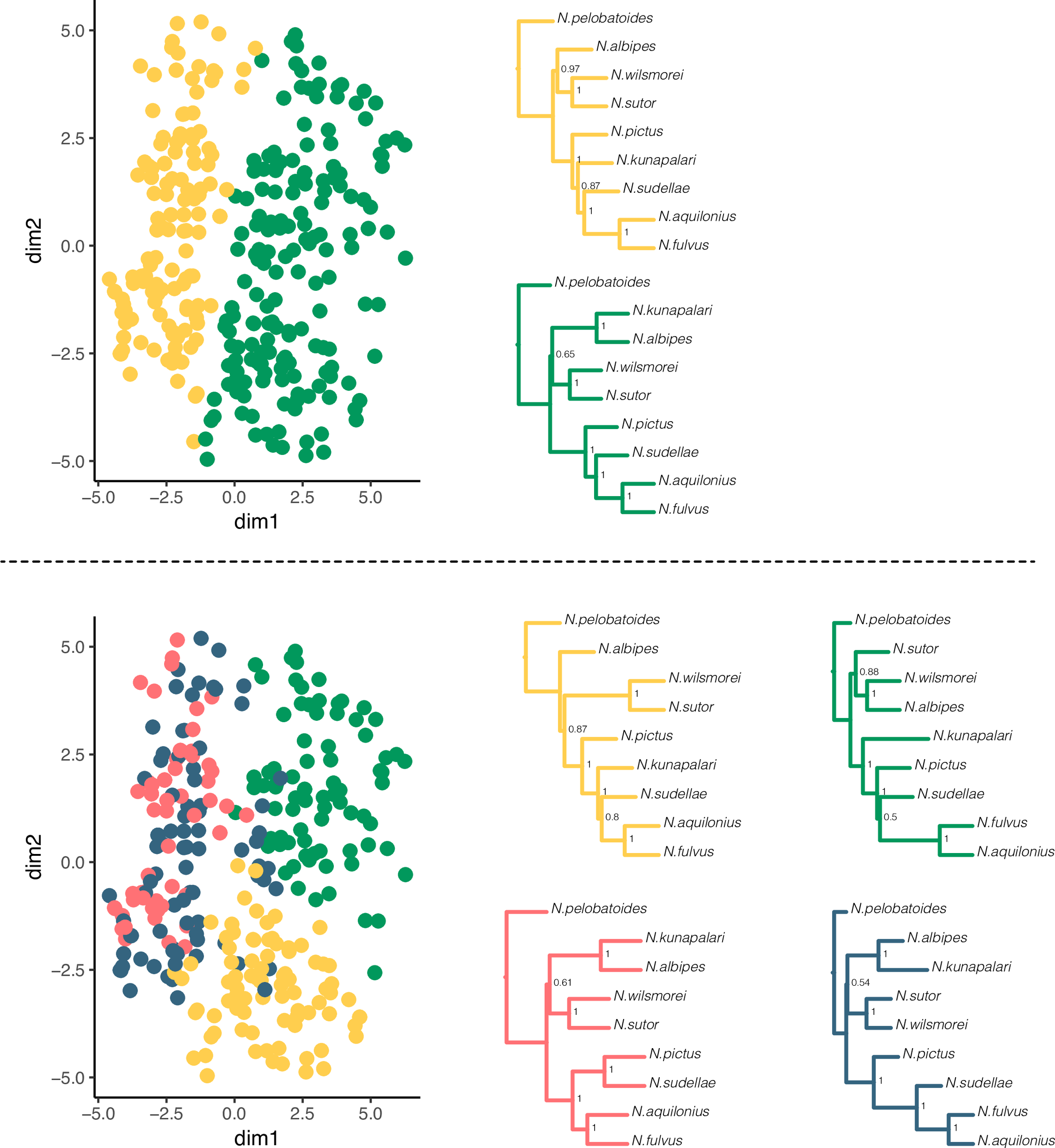

Supplement: S3 Fig — Each point represents a single gene tree, colored clusters match colored trees displayed to the right. Nodes at values indicate bootstrap support. (TIF) [file pgen.1008769.s008.tif]

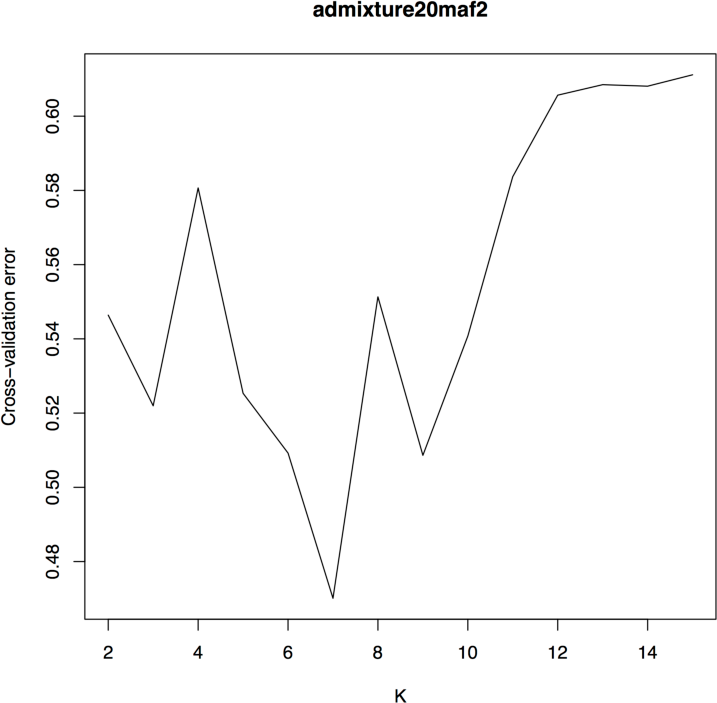

Supplement: S4 Fig — (TIF) [file pgen.1008769.s009.tif]

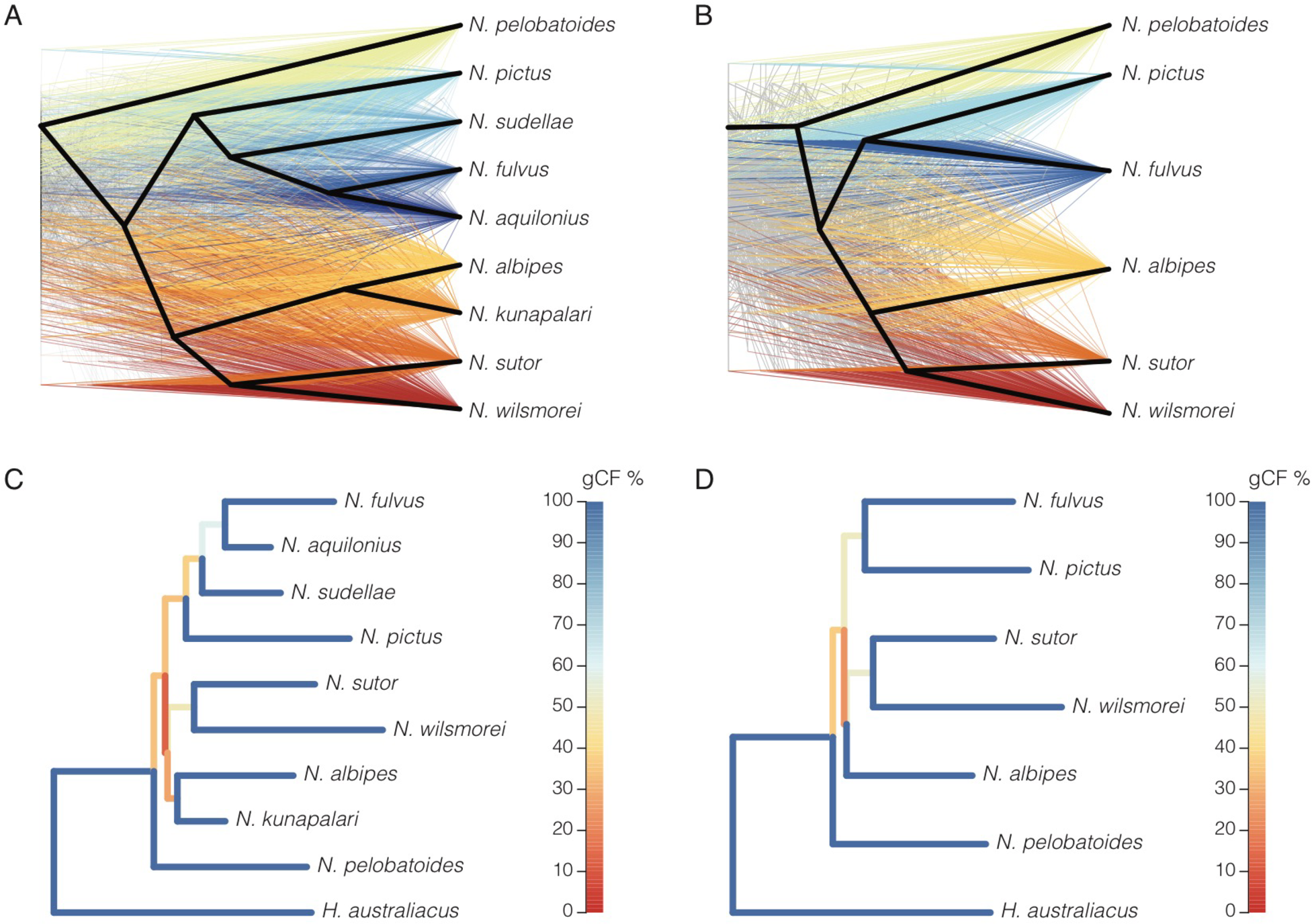

Supplement: S5 Fig — (A) Gene trees, colored by clade, for 361 nuclear loci based on 2 individuals per species show considerable incongruence and differ from the species trees (bold black topology). (B) Gene trees for diploid individuals only also show considerable incongruence and differ from the species trees (bold black topology). (C,D) Species tree colored by topological consistency as measured by gene concordance factors—gCF%, the percentage of loci which decisively favor a given bipartition. Warmer colors indicate high discordance, cooler colors indicate strong concordance. (TIF) [file pgen.1008769.s010.tif]

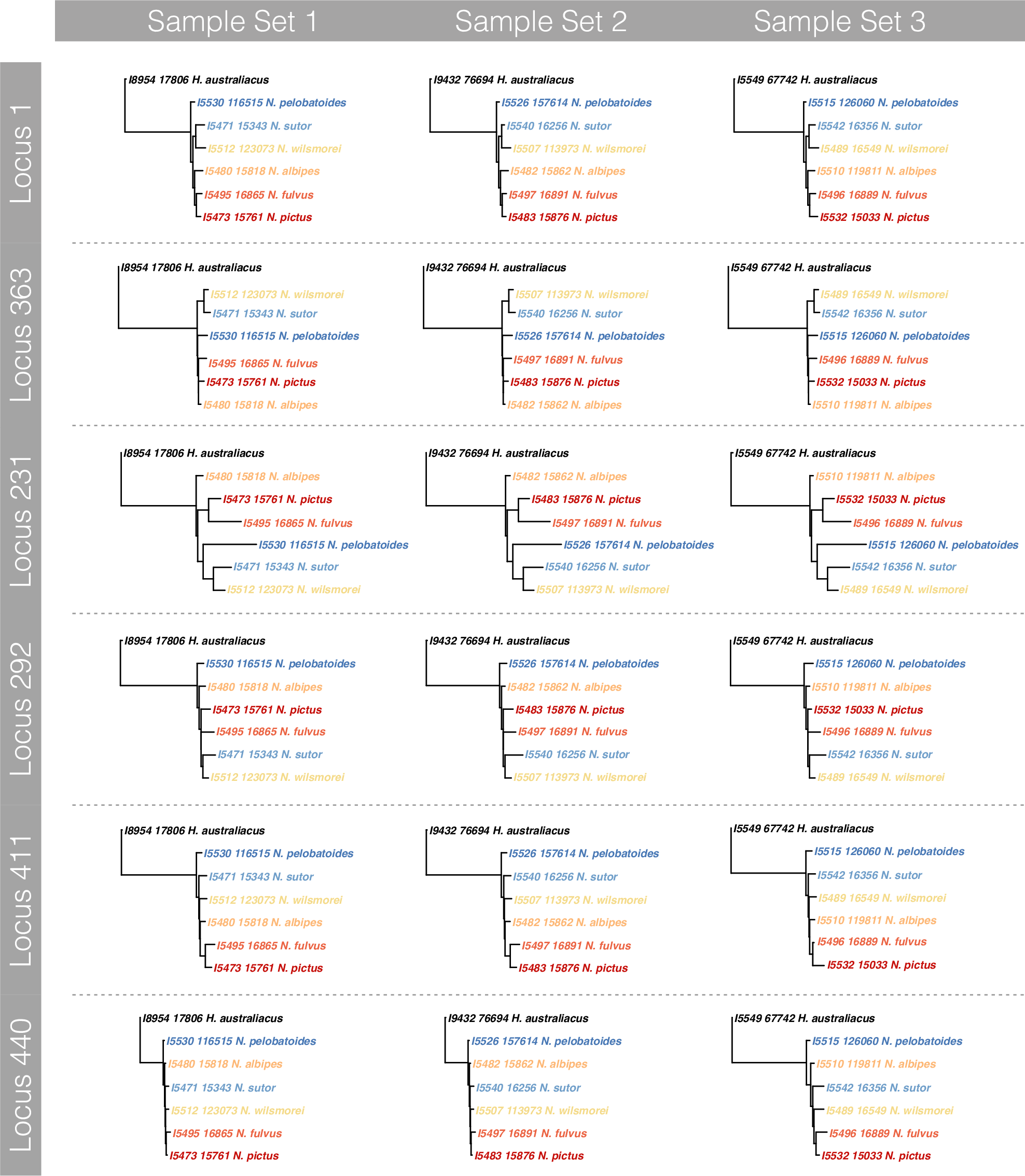

Supplement: S6 Fig — Genealogical conflict remains only among loci. This supports a scenario of rapid speciation of the diploid species without secondary contact or persistent incomplete lineage sorting. (TIF) [file pgen.1008769.s011.tif]

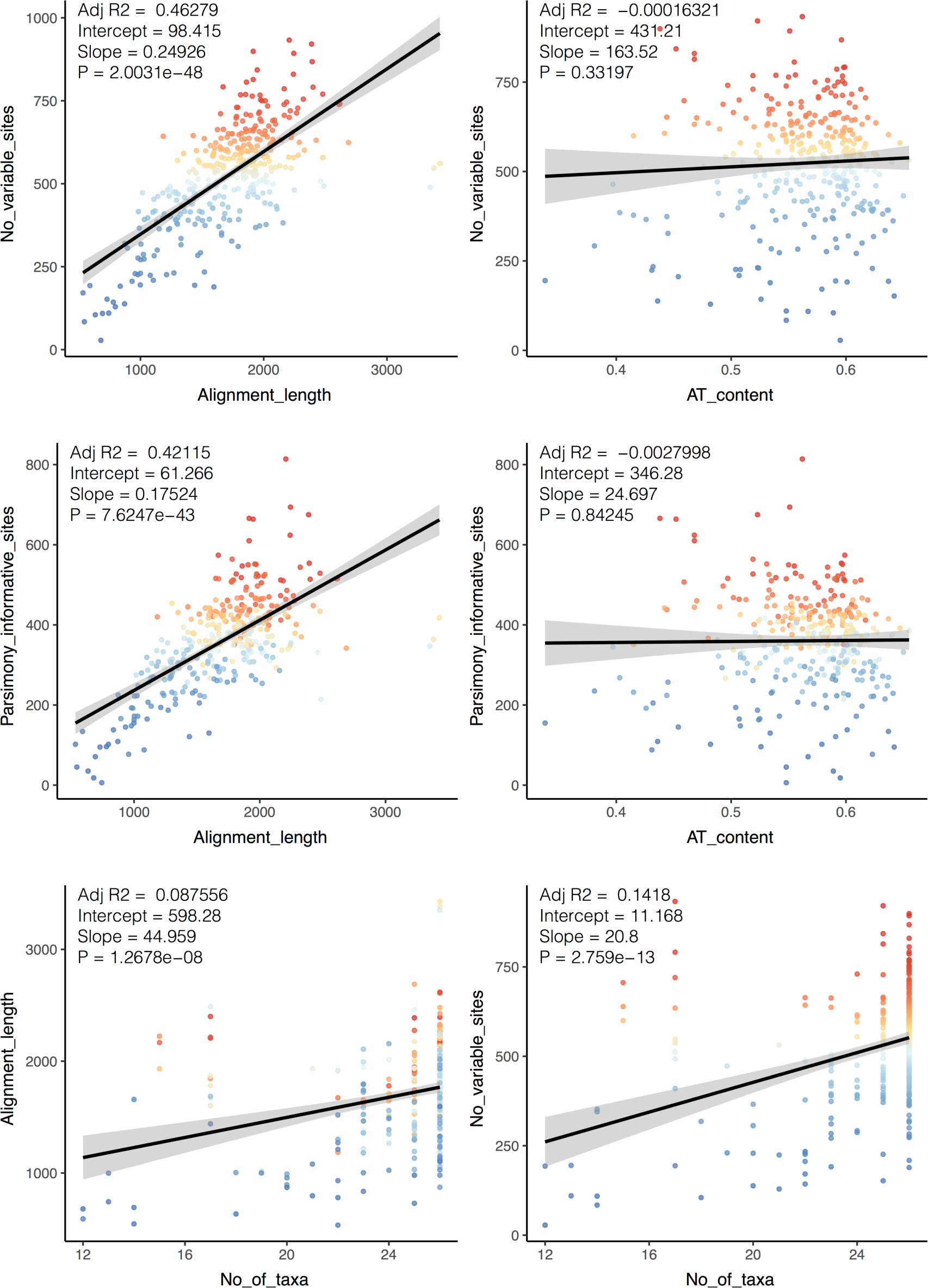

Supplement: S7 Fig — (TIF) [file pgen.1008769.s012.tif]

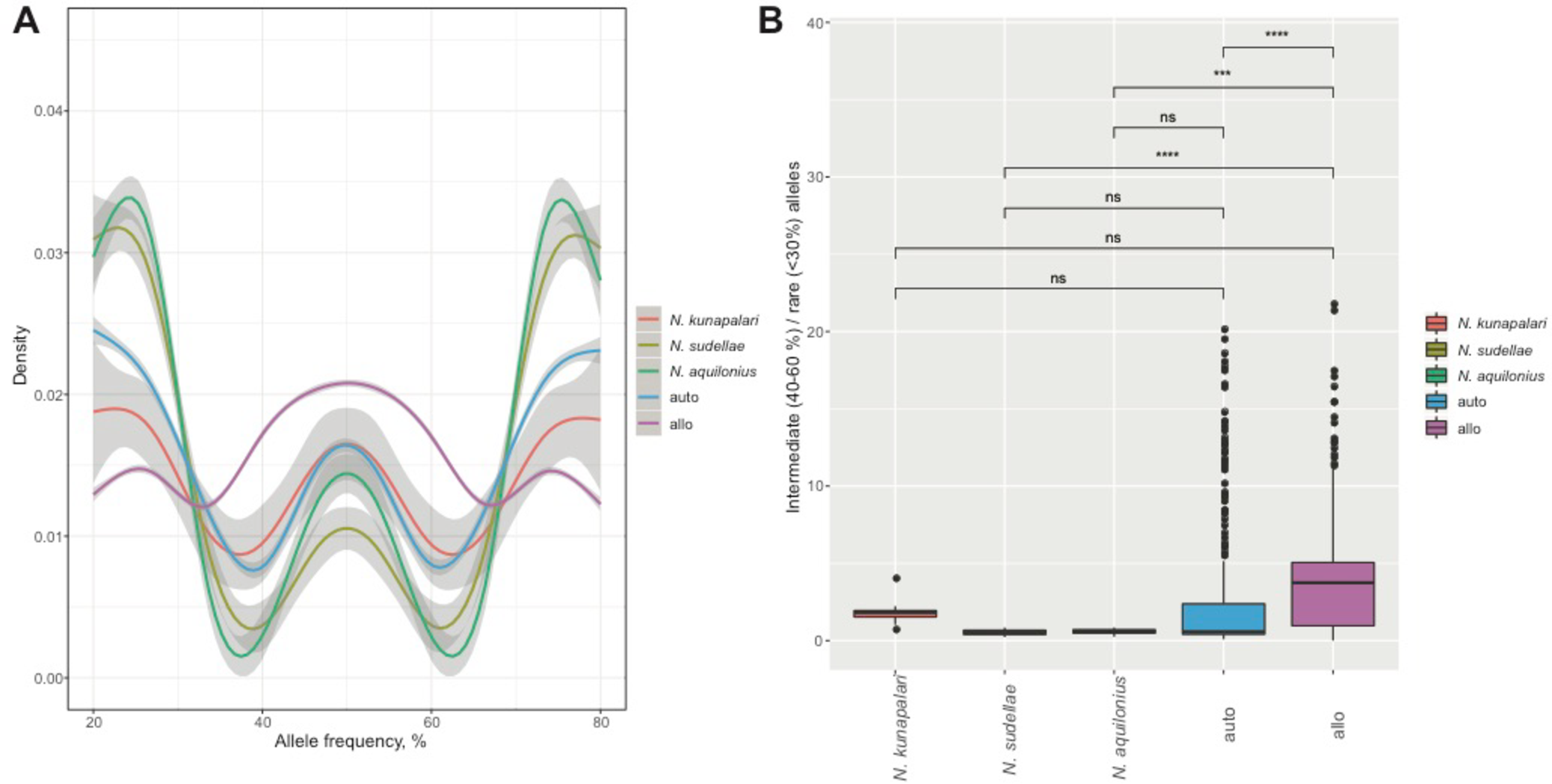

Supplement: S8 Fig — (A) Pairwise combination of individuals within the diploid species model the expected allele frequencies in autotetraploids with tetrasomic inheritance (blue line), when pairwise combination of individuals between the diploid Neobatrachus species model the expected distribution for allotetraploids with disomic inheritance mode (purple line). Modeled allotetraploids show excess of intermediate allele frequencies compared to autotetraploids. Gray area shows 95% confidence interval. (B) Comparing the ratio between intermediate (40–60%) and rare (<30%) allele frequencies we reject allotetraploid origin for N. sudellae and N. aquilonius, when N. kunapalari shows intermediate distribution, suggesting mixed inheritance. Comparisons performed with Wilcoxon tests adjusted for multiple testing. (TIF) [file pgen.1008769.s013.tif]

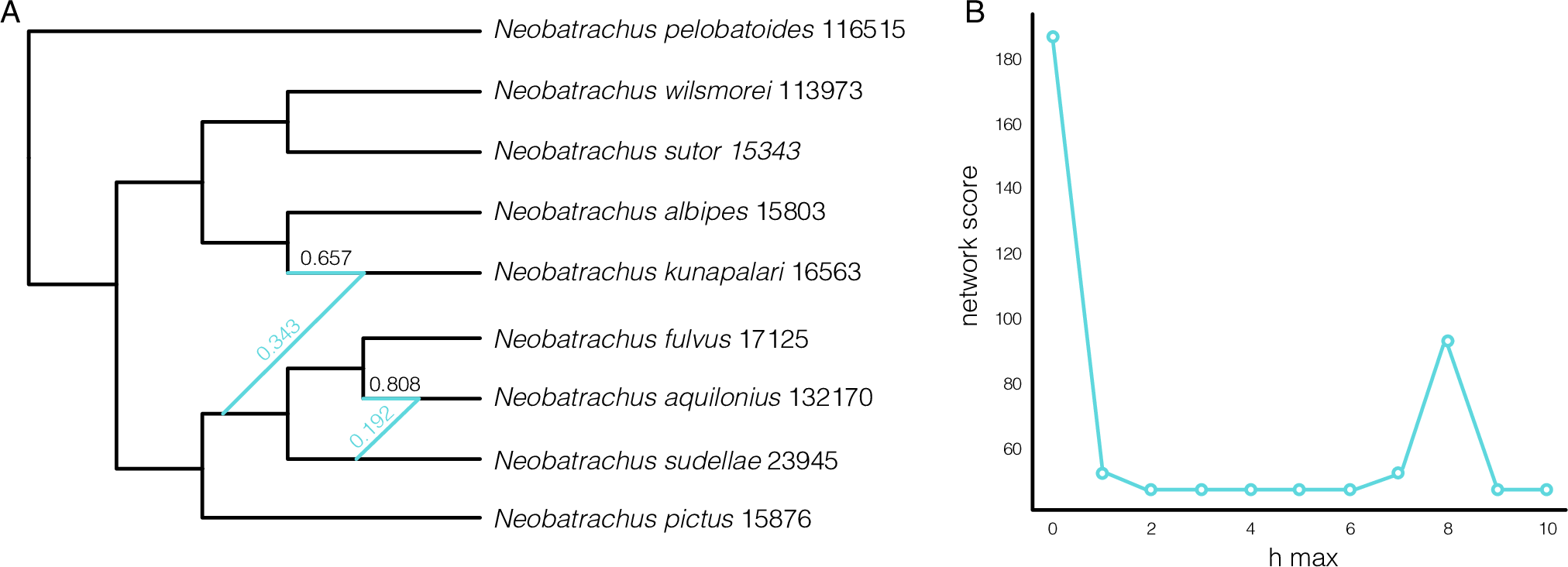

Supplement: S9 Fig — A. The optimum phylogenetic network includes two hybridization events. B. Network score has the best support at minumum 2 hybridization events, additional allowed hybridizations do not increase the network score. (TIF) [file pgen.1008769.s014.tif]

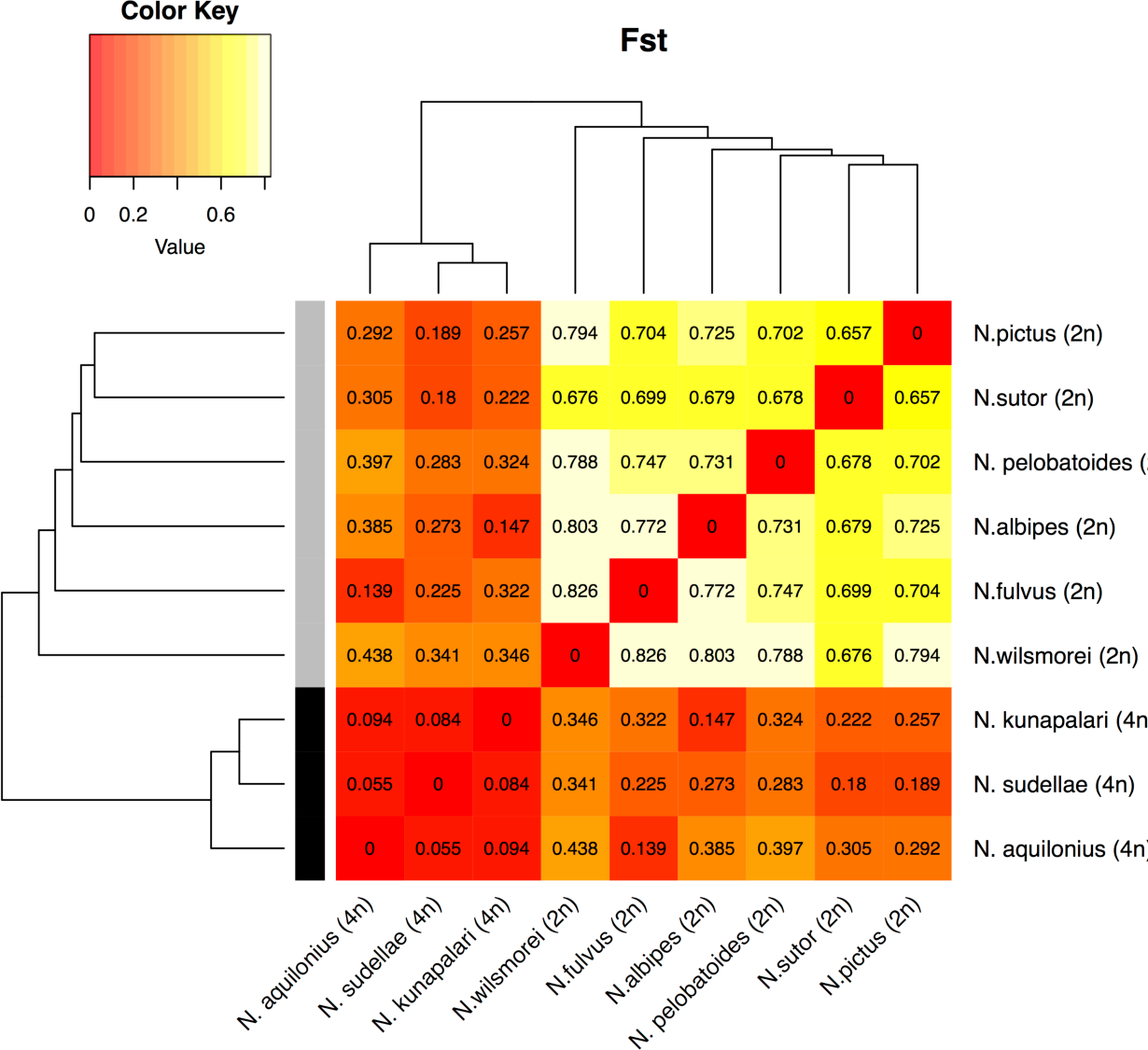

Supplement: S10 Fig — Tetraploid species (N. sudellae, N. aquilonius and N. kunapalari; highlighted with black left bar) cluster together and are characterised by the lowest Fst values between each other. This, together with low Fst values between tetraploid and diploid lineages, can probably be explained by the gene flow within the tetraploids and between the diploids and the tetraploids. Diploid lineages (highlighted with grey left bar) appear to be more isolated from each other compared to tetraploids, which is in agreement with ADMIXTURE assignment results and TreeMix estimations of possible migration events. (TIF) [file pgen.1008769.s015.tif]

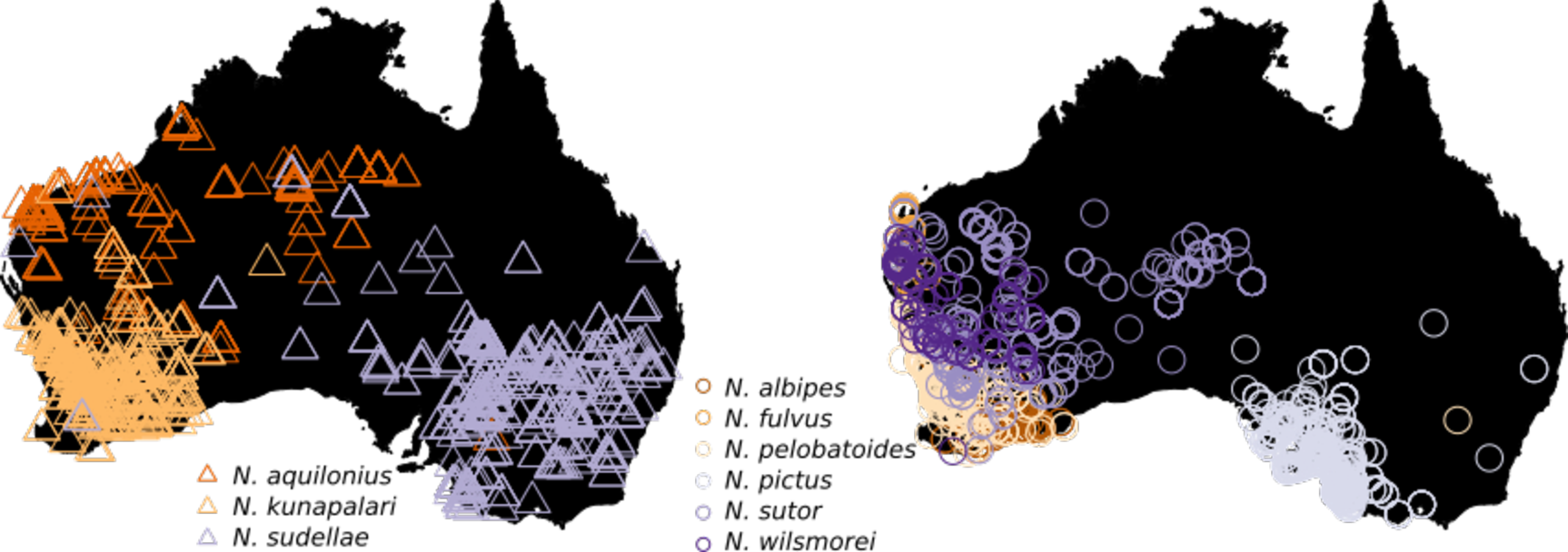

Supplement: S11 Fig — (TIF) [file pgen.1008769.s016.tif]

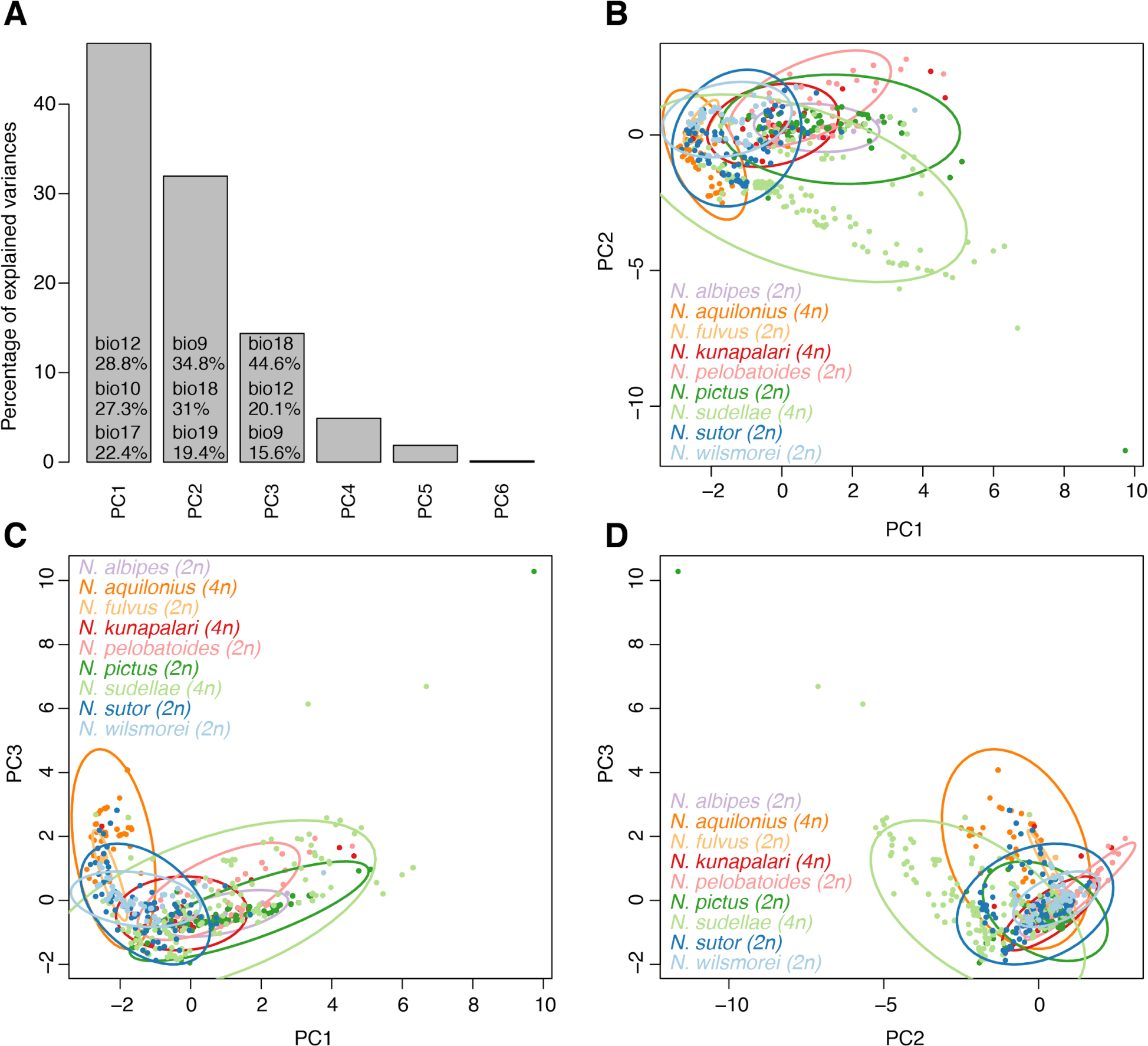

Supplement: S12 Fig — A) Barplot showing the percentage of variances explained by each principal component. The first three principal components are labeled with the top three contributions of variables. BIO10 = Mean Temperature of Warmest Quarter, BIO12 = Annual Precipitation, BIO17 = Precipitation of Driest Quarter, BIO18 = Precipitation of Warmest Quarter, BIO19 = Precipitation of Coldest Quarter. B-D) Pairwise combinations of the first three principal components, where individuals with a similar profile of bioclimatic data are grouped together. Points represent each individual and colored according to the species assignment, ellipses represent 95% confidence area. (TIF) [file pgen.1008769.s017.tif]

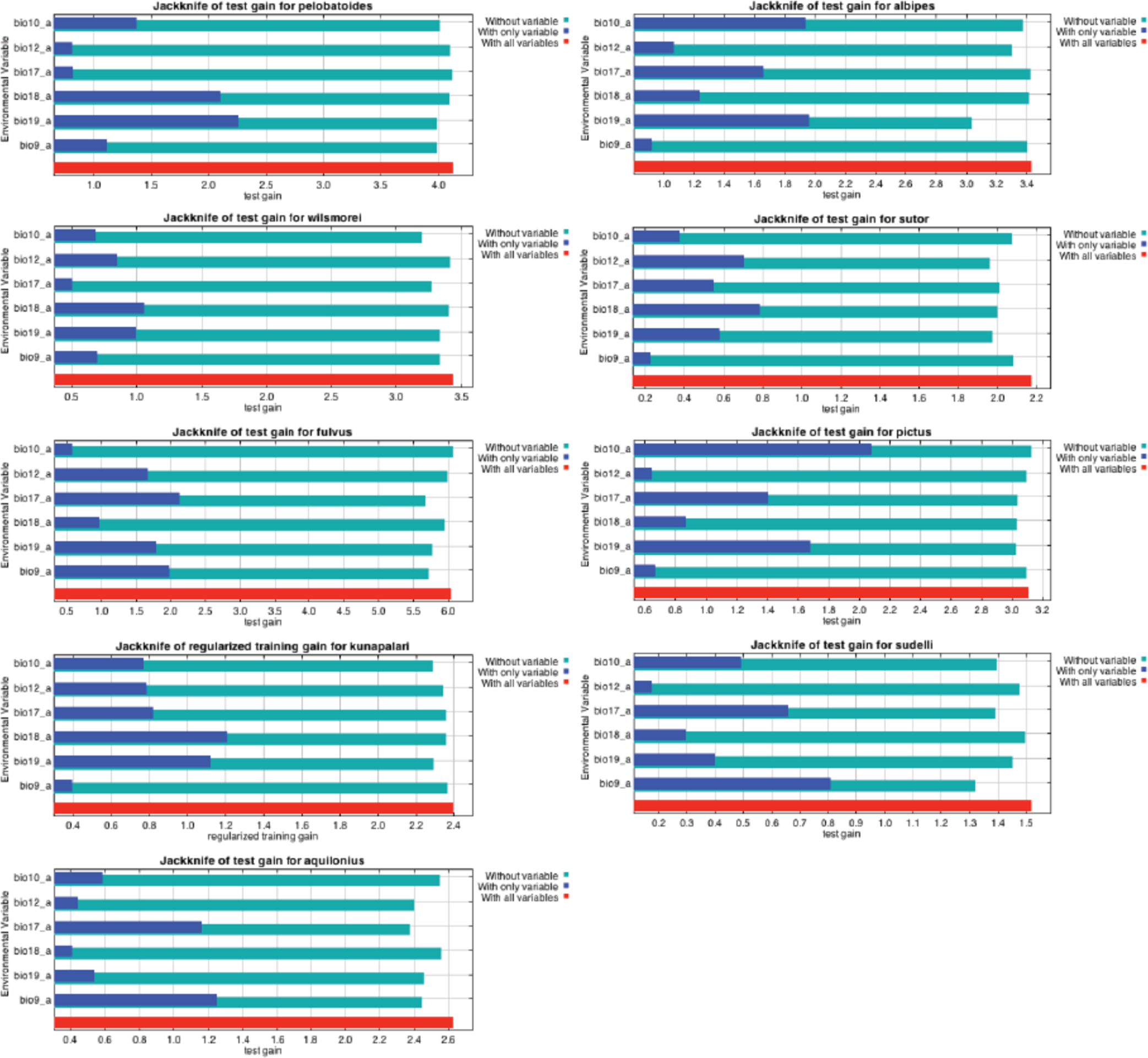

Supplement: S13 Fig — BIO19 (Precipitation of Coldest Quarter) was the most informative variable for the models of N. pelobatoides and N. albipes distributions; BIO18 (Precipitation of Warmest Quarter) was the most informative variable for the models of N. wilsmorei, N. sutor and N. kunapalari; BIO17 (Precipitation of Driest Quarter) was the most informative variable for the model of N. fulvus; BIO10 (Mean Temperature of Warmest Quarter) for N. pictus; and BIO9 (Mean Temperature of Driest Quarter) for N. sudellae and N. aquilonius. (TIF) [file pgen.1008769.s018.tif]

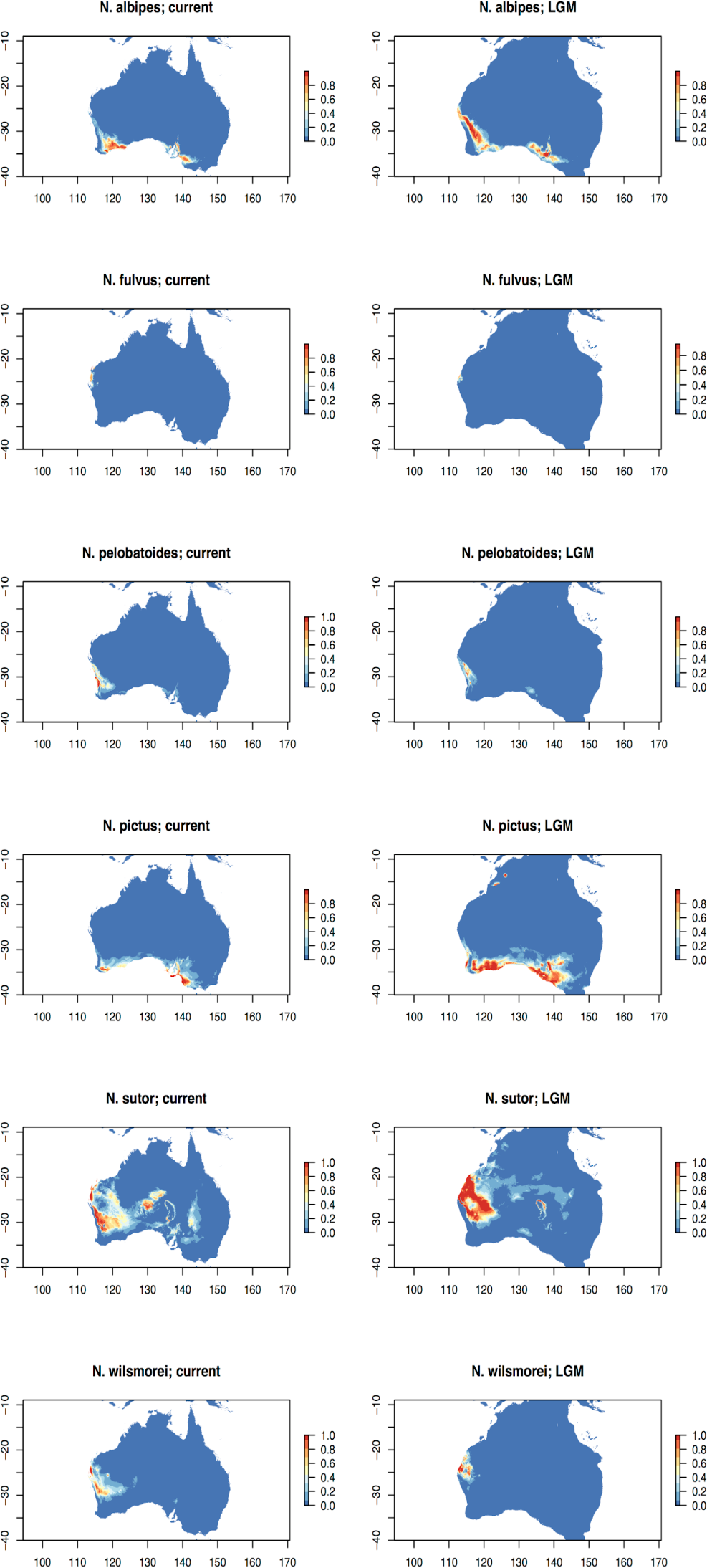

Supplement: S14 Fig — (TIF) [file pgen.1008769.s019.tif]

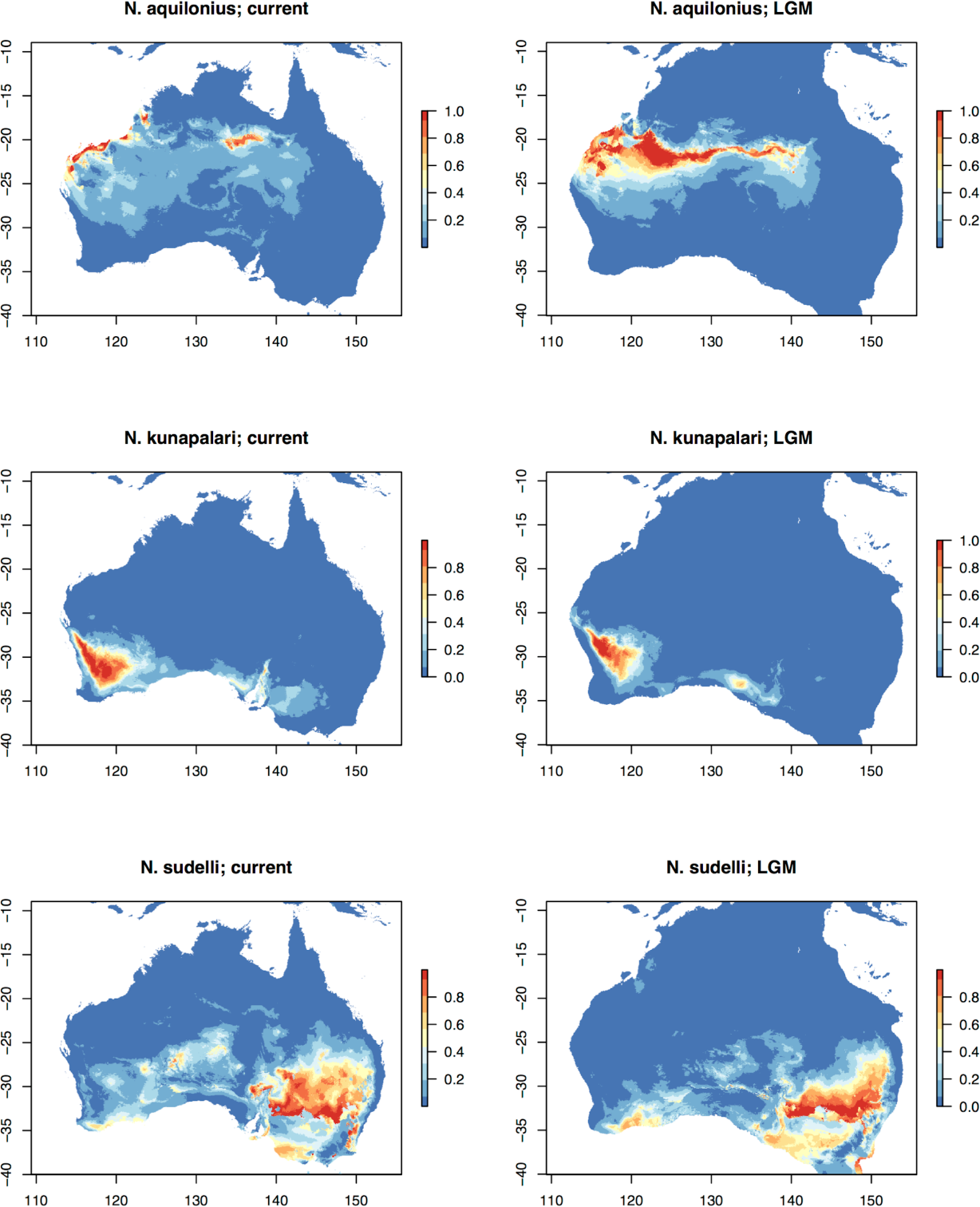

Supplement: S15 Fig — (TIF) [file pgen.1008769.s020.tif]

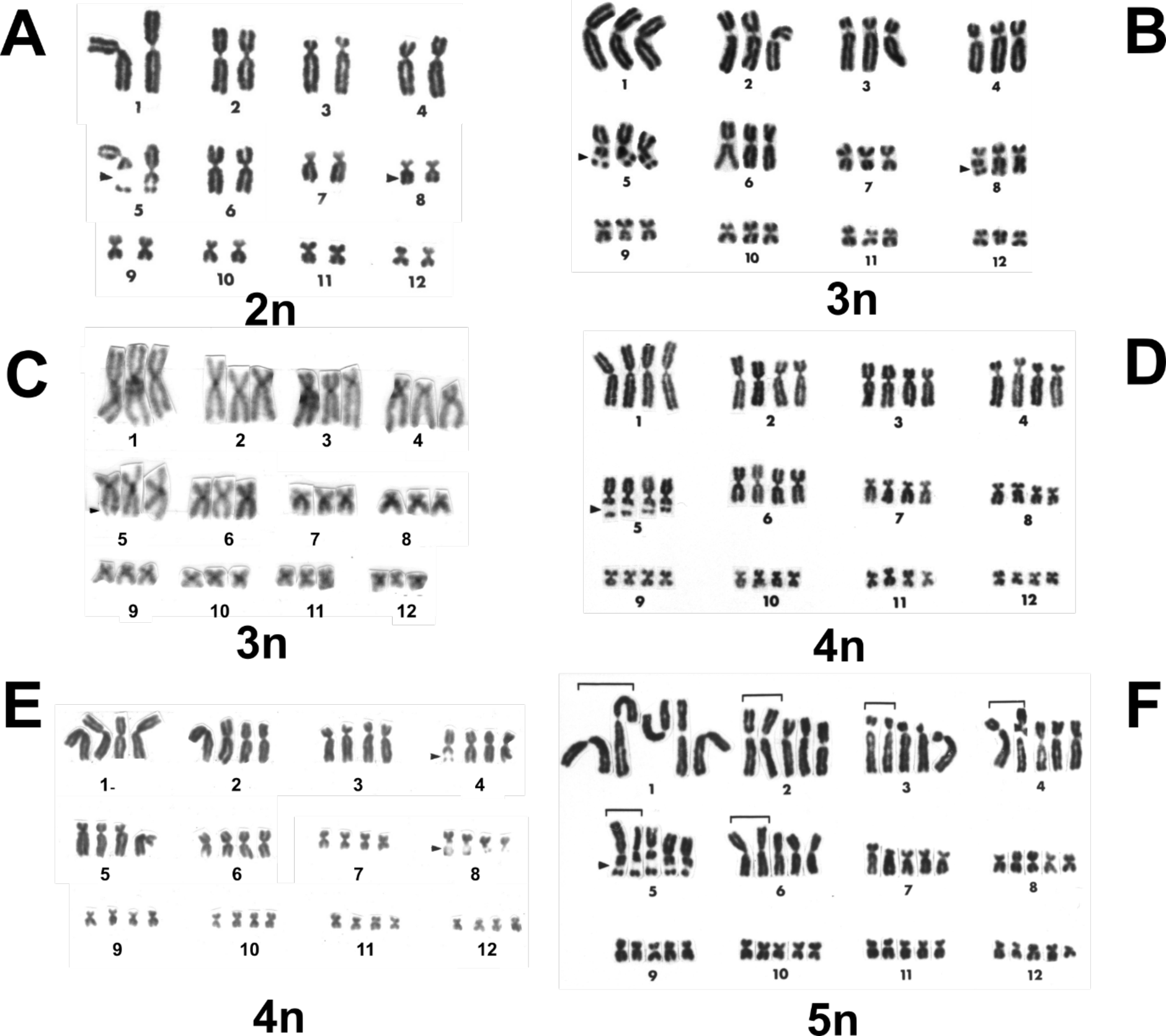

Supplement: S16 Fig — A) N. sutor [2n], B) N. pictus x N. sudellae triploid [3n] hybrid from Moyston, east of the Grampians, Victoria, C) N. fulvus x N. sutor triploid [3n] hybrid from Learmonth, Western Australia, D) N. sudellae [4n], E) tetraploid x tetraploid hybrid from north of Menzies, Western Australia, F) N. pictus x N. sudellae pentaploid [5n] hybrid from Moyston, east of the Grampians, Victoria. Arrowheads indicate nucleolar organiser regions (NORs). (TIF) [file pgen.1008769.s021.tif]
